# Silica Nanoparticles from Sustainable Sources: Fundamentals of Processing and Emerging Strategies

**DOI:** 10.3390/gels12090759

**Published:** 2026-08-24

**Authors:** Awadh O. AlSuhaimi, Khaled M. AlMohaimadi

**Affiliations:** 1 Department of Chemistry, Faculty of Science, Taibah University, AlMedina AlMunawarah 42367, Saudi Arabia; 2 Ministry of Education, Director General of Education, AlMedina AlMunawarah 42314, Saudi Arabia

**Keywords:** sustainable silica synthesis, silica nanoparticles, agricultural waste, rice husk ash, fly ash, geothermal silica, fluorosilicic acid, green chemistry, ambient-pressure drying, feedstock impurities, energy balance, life cycle assessment, circular economy, structure–property trade-off, emerging strategies

## Abstract

The transition from conventional silica nanoparticle (SiNP) production based on purified alkoxysilanes and high-temperature flame hydrolysis of silicon tetrachloride to renewable and waste-derived silicon resources requires more than precursor substitution. It requires a mechanistic understanding of how feedstock mineralogy, silicon speciation, impurity chemistry, and processing history propagate through dissolution, nucleation, condensation, gelation, aging, drying, and pore evolution to determine material performance, environmental burden, and manufacturing feasibility. Although previous reviews have established the technical feasibility of producing silica from secondary resources, their predominant organization by feedstock, synthesis route, or application provides limited ability to explain why nominally similar processes generate materials with markedly different structural and functional properties. This review addresses these through a resource-pull, feedstock-to-function framework that links resource chemistry and process design to critical material attributes, application-specific specifications, sustainability, and scale-up requirements. Agricultural residues, industrial by-products, geothermal resources, waste glass, and fluorosilicate streams are critically compared according to silicon form and phase, reactivity, impurity profile, compositional variability, purification demand, and attainable product quality. Particular attention is given to waste-derived alkaline silicate systems, in which molecular, oligomeric, and colloidal silica coexist and therefore require characterization beyond bulk SiO_2_ concentration. Established and emerging processing strategies, including controlled combustion and alkaline extraction, alkali fusion, ambient-pressure drying, microwave and mechanochemical activation, biogenic and biomimetic templating, and continuous processing, are evaluated according to their mechanistic effects, technological maturity, structural control, and demands for energy, reagents, water, solvents, effluent treatment, and capital. Across these routes, gelation and aging emerge as critical transfer stages through which feedstock composition is translated into network connectivity, pore architecture, shrinkage behavior, and ultimately functional performance. Evidence from secondary-source aerogels further shows that properly controlled waste-derived systems can attain BET surface areas of approximately 350–500 m^2^ g^−1^, within the textural range of many alkoxide-derived materials, indicating that feedstock variability, impurity management, and process control are more important constraints than an inherently lower performance ceiling. On this basis, this review proposes a minimum evidence framework comprising feedstock traceability, intermediate-speciation and colloidal characterization, silicon mass balance, gelation and aging metrics, application-specific qualification criteria, performance-normalized life cycle and techno-economic assessment, process analytical control, and staged pilot validation. Collectively, these principles provide a mechanistically grounded basis for moving sustainable silica synthesis beyond isolated proof-of-concept demonstrations toward reproducible, scalable, application-matched, and commercially credible manufacturing platforms.

## 1. Introduction

Silica nanoparticles (SiNPs) comprise dense colloids, porous particles, gels, aerogels, coatings, aggregates, and hybrid structures whose performance is governed by a small set of coupled attributes: external size and aggregation state, pore architecture, network condensation, and surface silanol chemistry [1,2,3]. These attributes determine transport, molecular accessibility, dissolution, interfacial reactivity, functionalization efficiency, and ultimately application performance. A sustainable route should therefore be judged by whether it reproduces the critical attributes required for a defined function, rather than by nominal SiO_2_ purity alone.

Silica formation follows a common network-building chemistry, but the point at which a precursor enters that chemistry depends strongly on its chemical form. Alkoxysilanes first undergo water-mediated hydrolysis of Si–OR groups to generate silanol-rich species, whereas aqueous alkali silicates already contain hydrated and deprotonated molecular, oligomeric, and, in some formulations, colloidal silicate/silica populations. Dilution and acidification of water glass shift hydrolysis/protonation equilibria toward silicic acid, Si(OH)_4_, while also changing colloidal stability. The resulting Si–OH-bearing species then undergo condensation/polymerization, nucleation or aggregation, gelation, aging, and drying [4,5,6,7,8]. Sustainable feedstocks therefore do not create a separate silica chemistry; rather, their silicon speciation, ionic strength, residual carbon, trace metals, and pre-existing colloidal fraction alter the starting state and the kinetics through which the common Si–O–Si network is formed.

Recent reviews have established the technical feasibility of green synthesis, agro-waste extraction, geothermal silica recovery, waste-derived mesoporous materials, and application-specific systems [1,9,10,11,12,13,14,15,16,17,18]. The unresolved need is mechanistic integration. Two materials reported at the same nominal purity may differ in Q^2^/Q^3^/Q^4^ connectivity, colloidal fraction, residual alkali, pore accessibility, dissolution kinetics, and biological response. This review addresses that gap by treating sustainability as a property of the complete feedstock–process–material–application system and by linking resource chemistry directly to process control and qualification.

### 1.1. Unique Properties and Cross-Application Design Variables of SiNPs

The most transferable descriptors for cross-application design are particle size distribution in the relevant medium, accessible surface area and pore size distribution, network connectivity, and surface chemistry. Their relative weighting shifts with the intended use: adsorption and catalysis place primary emphasis on accessible interfaces and mass transport; coatings and composites depend critically on dispersion quality and interfacial bonding; pharmaceutical systems demand simultaneous control of particle size distribution, surface state, dissolution kinetics, and impurity burden [1,2,3].

Biomedical translation imposes the most rigorous test of this framework. Particle size and aggregation state govern vascular transport and phagocytic recognition; pore architecture dictates loading capacity and release kinetics; surface silanol density and grafted ligands determine protein adsorption and membrane interactions; framework connectivity controls dissolution rate and clearance pathways [19,20,21,22,23,24]. Waste-derived origin does not relax these requirements. Any biomedical claim further necessitates feedstock traceability, elemental impurity control, residual template and solvent quantification, sterility or bioburden assessment, endotoxin control where applicable, colloidal stability in the administration medium, and demonstrated batch-to-batch comparability. Regulatory constraints therefore function as design inputs that must be incorporated before a synthesis route is selected, rather than applied retrospectively after material optimization.

### 1.2. The Evolution of Silica Science and the Emergence of Sustainable Production

Colloid and sol–gel chemistry first quantified the relationships among silicate speciation, condensation, gelation, aging and drying [4,5,6,7,8]. The Stöber process then showed that controlled hydrolysis–condensation of alkoxysilanes could dictate nucleation and growth kinetics, yielding monodisperse spheres [25]. Concurrent work on the M41S and SBA-15 families established that surfactant-directed assembly allows pore diameter, wall thickness and mesostructure to be adjusted independently of particle size [26,27,28]. These mechanistic foundations remain the benchmark against which every sustainable silica route is judged.

Sustainable routes apply identical chemistry to compositionally heterogeneous silicon sources—agricultural residues, industrial by-products, geothermal brines and fluorosilicate streams [10,11,12,29,30,31]. Each feedstock carries its own mineral phases, impurity profiles and dissolution energetics, which in turn shift the condensation pathway and constrain the attainable purity, surface chemistry and process intensity. The scientific task is therefore to map how feedstock composition modifies reaction trajectory and to define the product specifications that can still be reached under realistic process constraints.

### 1.3. Contemporary Drivers for Sustainable Silica Production

The chemistry governing silica formation remains unchanged, yet the conditions governing silicon supply have shifted. The demand for high-purity silicon-bearing feedstocks continues to rise while economically viable deposits of high-purity quartz remain limited in both quantity and accessibility. The conversion of lower-grade quartz into usable material requires intensive mineral liberation, acid leaching, substantial energy input and prolonged processing times [29,31,32]. In parallel, large agricultural and industrial residue streams contain recoverable silicon that can be processed into alternative silica precursors. The simultaneous pressure of constrained primary resources and available secondary silicon increases the technical attractiveness of residue-based routes. Residue availability by itself does not establish a lower environmental burden: the complete sequence of collection, pretreatment, purification and product specification must still be quantified and compared directly with primary production routes.

**Figure 1:**
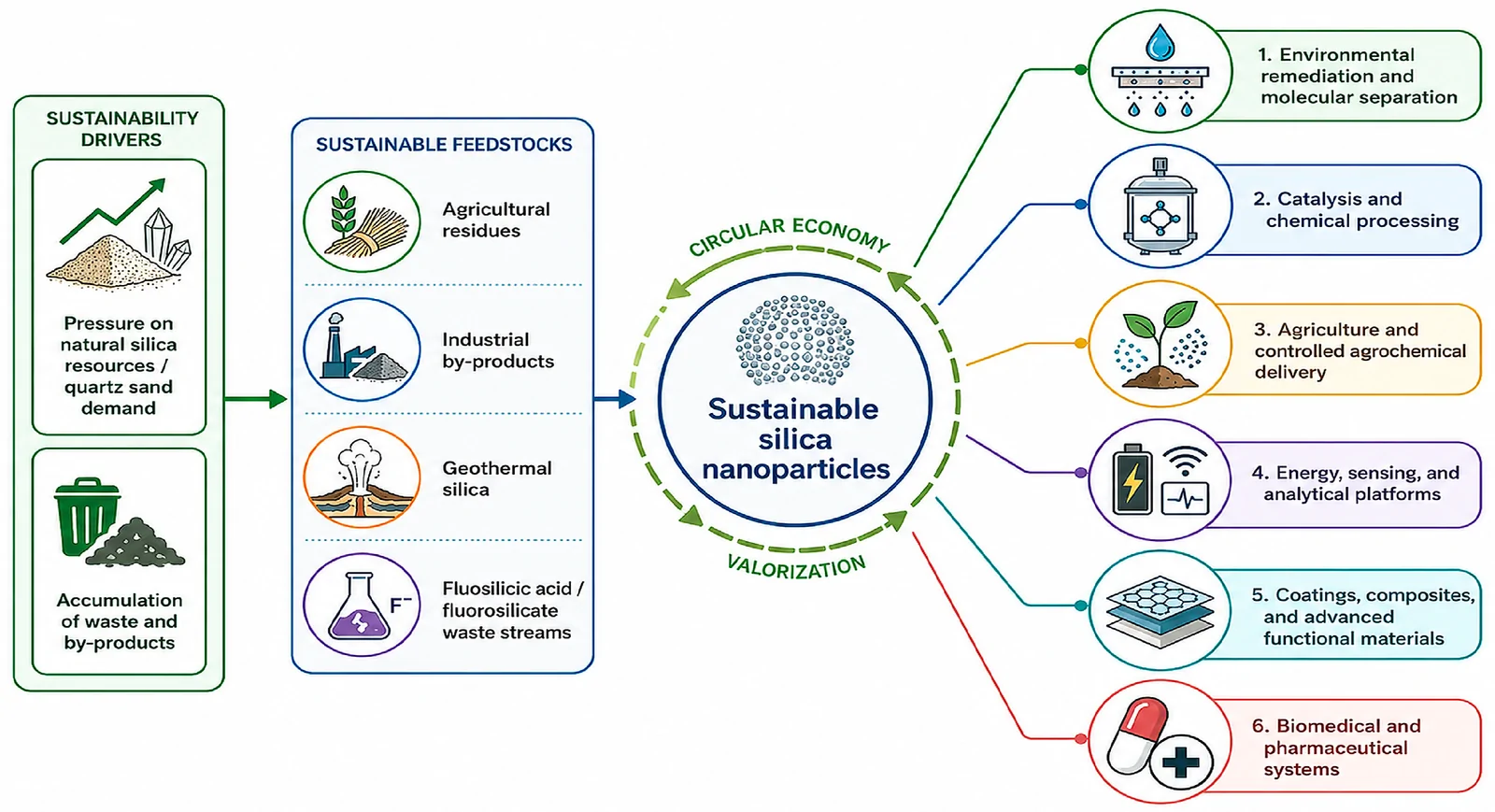
Sustainability drivers for alternative silica production pathways. Pressure on natural silica resources and the accumulation of agricultural residues, industrial by-products, geothermal precipitates, and fluorosilicate wastes create parallel incentives for secondary silica recovery. Valorization can reduce disposal burdens and dependence on virgin minerals, but the net benefit depends on collection, pretreatment, purification, energy source, product quality, and the functional value of the resulting silica nanoparticle.

The convergence of these trends has created strong incentives to develop circular pathways for silica production from secondary resources. Agricultural residues such as rice husk ash and sugarcane bagasse ash, industrial by-products including fly ash and slag, geothermal silica deposits, and fluorosilicic acid generated during phosphate fertilizer production all contain significant quantities of recoverable silicon [10,11,12,13,14]. Converting these materials into functional silica products aligns with circular economy principles by simultaneously reducing waste disposal burdens and decreasing dependence on virgin mineral resources. Figure 1 summarizes these interacting drivers and shows that the case for alternative silica production depends on both resource pressure and the practical conditions under which secondary silicon streams are recovered and processed.

### 1.4. Existing Reviews, Technology-Push Bias, and the Unresolved Research Gap

A substantial body of reviews document the conversion of renewable feedstocks, agricultural residues, industrial by-products, geothermal silica and related secondary sources into nanostructured silica [1,9,10,11,12,13,14,15,16,17,18]. Two conclusions emerge: secondary silicon resources are chemically accessible, and many can be transformed into materials that exhibit useful surface area, porosity and functionality. The outstanding difficulty is not a shortage of examples, but the absence of a common explanatory framework that renders those examples comparable and that accounts for why apparently similar routes generate systematically different materials.

Most published reviews retain a technology-push organization. They begin with a familiar product or synthesis route, identify a waste stream capable of supplying silicon, and judge success by the extent to which the conventional material can be reproduced. This approach has established technical feasibility, yet it produces catalogs of positive endpoints rather than causal accounts of process behavior. Feedstock origin functions primarily as a label; the chemical form of silicon, the covariance of impurities, prior thermal history and the conditions used to generate the reactive silicate solution receive uneven attention. As a result, the literature contains numerous successful syntheses but few transferable design rules.

There are five linked gaps that still limit progress. First, mechanistic understanding remains incomplete. Waste-derived silicate solutions are commonly characterized by nominal SiO_2_ content or final purity, although aluminum, calcium, magnesium, sodium, potassium, iron, residual carbon and trace elements alter silicate speciation, ionic strength, condensation kinetics, colloidal stability, gelation, aging and pore development [10,12,33,34,35,36,37]. These species are typically quantified after synthesis as residual contaminants rather than investigated prospectively as kinetic and structural variables. Controlled concentration–response studies, including multicomponent impurity interactions, remain scarce. No general model yet predicts how a measured feedstock composition will modify gel time, Q^n^ connectivity, particle size distribution or pore network evolution.

Second, the meaning of sustainable processing is under-specified. A waste precursor, lower reaction temperature, or shorter synthesis time is often treated as sufficient evidence of improvement, while burdens are shifted to alkali production, acid neutralization, repeated washing, solvent exchange, silylation, drying, corrosion-resistant equipment, or wastewater treatment. Life cycle comparisons are especially sensitive to the functional unit, system boundary, allocation of waste and avoided-disposal credits, electricity mix, transport distance, reagent recovery, product yield, and whether off-specification material is included [9,10,38,39,40]. Circularity claims require the same care because valorizing a residue can redistribute burdens rather than eliminate them; waste-derived silica used for fly ash inertization provides a concrete example, while broader circular economy frameworks emphasize system-level trade-offs [41,42,43]. These methodological choices must be declared before a carbon-reduction percentage is interpreted as a property of a route.

Third, equivalence and reproducibility remain poorly defined. Waste-derived and alkoxide-derived silicas are frequently compared using a single endpoint such as nominal purity, BET surface area, mean particle size, or adsorption capacity. These descriptors do not resolve pore size distribution, external versus internal area, network condensation, residual ionic species, dispersion state, dissolution behavior, or batch-to-batch uncertainty. A material may therefore appear equivalent by one metric while remaining unsuitable for the intended function. The literature rarely distinguishes an intrinsic limitation of the feedstock from variability introduced by incomplete feedstock qualification, poorly controlled acidification, aging, washing, or drying.

Fourth, scale-up evidence is limited. Most studies are performed in small, well-mixed batch vessels where reagent addition, heat transfer, and sampling are readily controlled. At larger volumes, local pH and supersaturation gradients, mixing time, residence time distribution, heat and mass transfer, fouling, corrosion, and washing efficiency become determinants of nucleation and gelation. These variables are seldom reported, and continuous or semi-continuous demonstrations supported by process analytical technology remain exceptional. Laboratory success therefore provides limited evidence that a route will retain particle and pore control at manufacturing scale.

Fifth, translational requirements are treated as downstream activities rather than as inputs to process design. Purification, characterization, toxicological testing, and regulatory evidence are commonly added after the synthesis is fixed. This separation encourages maximum-purity targets even when the application does not require them, or, conversely, premature claims for biomedical, food, or agricultural use without impurity clearance, leaching, colloidal stability, dissolution, and exposure-relevant testing. The absence of application-specific critical quality attributes also prevents rational comparison between high-volume, impurity-tolerant products and low-volume products that require stringent compositional control.

The research gap addressed here is therefore specific and multi-level. The field still lacks an integrated, mechanistically grounded account that links feedstock mineralogy and composition to reactive silicate speciation, that then links speciation and process history to nucleation, gelation, network architecture and surface chemistry, and that finally evaluates the resulting material against performance-normalized environmental, economic, safety and scale-up criteria. Without this continuity it is not possible to identify which limitations are inherent to a secondary silicon resource, which arise from avoidable process control failures, and which are simply consequences of applying an unnecessarily stringent or poorly defined product specification.

**Figure 2:**
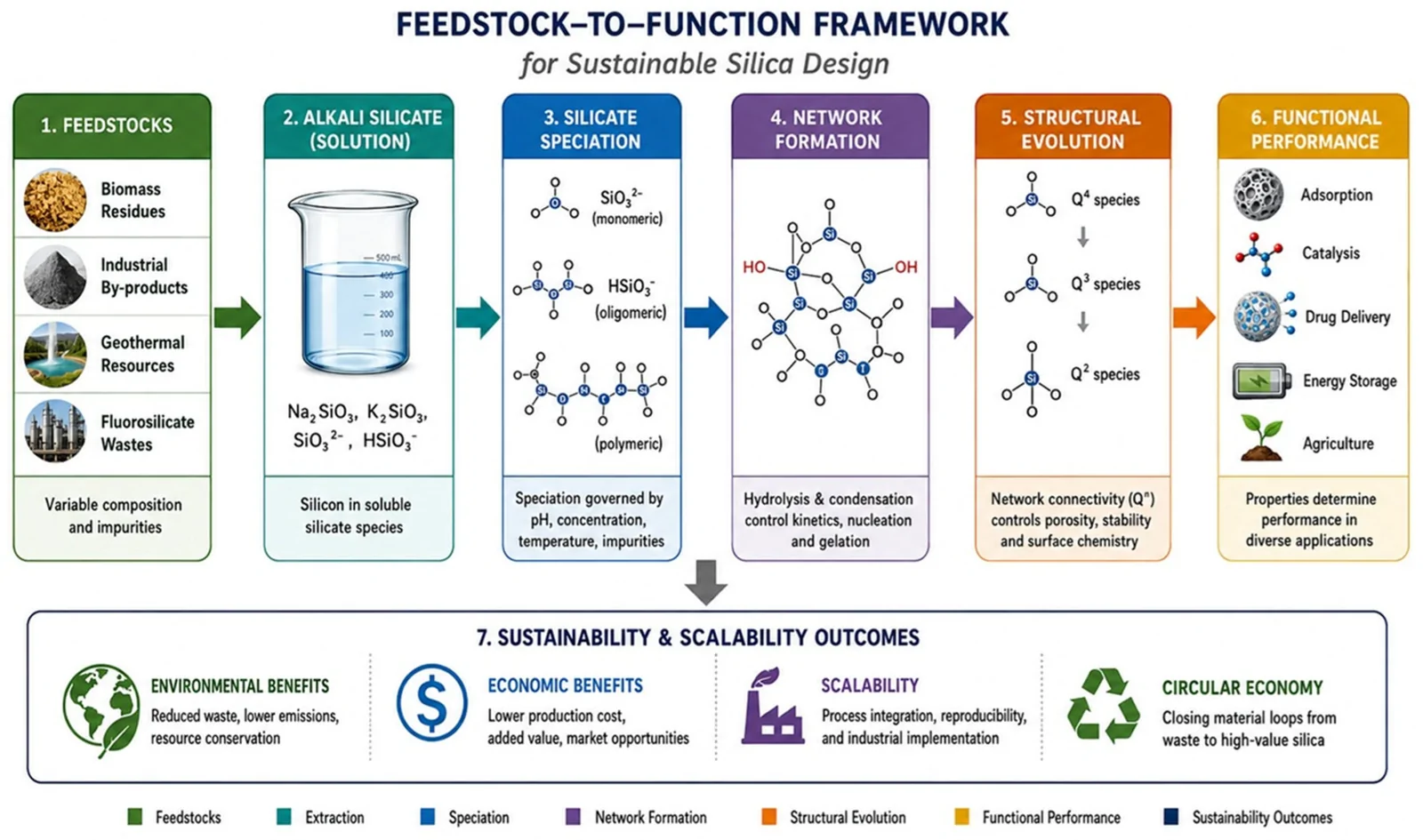
A feedstock-to-function framework for sustainable silica design. Feedstock mineralogy and impurities determine extraction efficiency and the composition of the resulting alkali silicate solution. Silicate speciation, condensation, network formation, and Q^n^ evolution then govern particle architecture, porosity, surface chemistry, and performance. Environmental benefit, economic feasibility, reproducibility, and scalability are treated as coupled outcomes of the complete pathway rather than intrinsic attributes of waste origin. Q^n^ denotes a silicon center connected through n bridging oxygens.

The present review addresses this gap by replacing the prevailing technology-push perspective with a resource-pull analysis (Figure 2). It begins with the chemical state and variability of the available silicon source and asks what product architectures and specifications that resource can support with defensible process intensity. The review is deliberately analytical rather than encyclopedic: it distills the developments that altered precursor and process selection, identifies the measurements needed to test causal relationships, and derives decision criteria for scale-up and application matching. The intended contribution is not another inventory of sustainable syntheses, but a framework for designing, comparing, and qualifying them.

### 1.5. Scope, Objectives, and Intended Readership

The scope is defined by the intersection of sustainable silicon sourcing and sustainable processing. Conventional sol–gel chemistry is used as the mechanistic reference, but mature theory is not reproduced in detail. Attention is directed instead to the points at which complex feedstocks and lower-impact processing alter reaction pathways, material attributes, reproducibility, and industrial or regulatory feasibility. Representative applications are included to test whether the proposed design principles remain valid across different impurity tolerances and performance requirements.

This review has six specific and actionable objectives. It intends to (i) construct a feedstock-to-function map that links silicon form, impurity composition, and processing history to silicate speciation, nucleation, gelation, Q^n^ connectivity, pore development, and surface reactivity; (ii) compare agricultural residues, industrial by-products, geothermal resources, waste glass, and fluorosilicate streams using common descriptors of availability, reactivity, purification demand, variability, and attainable specification; (iii) evaluate emerging processing strategies by the mechanisms through which they change energy, reagent, water, solvent, and effluent burdens while preserving structural control; (iv) distinguish intrinsic feedstock constraints from avoidable failures of mixing, acidification, aging, drying, washing, and quality control; (v) define a minimum evidence set for reproducible reporting, performance-equivalent comparison, life cycle and techno-economic assessment, scale-up, safety, and application qualification; and (vi) translate the synthesis evidence into concrete research priorities and decision rules for laboratory development, process optimization, and economically viable manufacturing. This review is intended principally for researchers in materials chemistry, sol–gel science, green chemistry, and nanomaterials engineering, while also serving process developers, industrial practitioners, sustainability and techno-economic analysts, and regulatory scientists.

The article is organized to connect fundamental chemistry with implementation. Section 2 establishes the minimum sol–gel framework required to interpret waste-derived systems, and Section 3 connects network formation to architecture and function. Section 4 and Section 5 develop the precursor and process axes, respectively. Section 6 and Section 7 define the characterization and interfacial evidence needed to verify material identity and performance. Section 8 compares application requirements by tolerance to feedstock variability, Section 9 evaluates life cycle, economic, and circularity claims, and Section 10 integrates performance comparison, standardization, scale-up, regulation, and detailed further directions before the conclusions in Section 11.

## 2. Sol–Gel Chemistry as an Established Platform and the Consequences of a Waste-Derived Precursor

The chemistry that converts reactive silicon species into a solid silica network is mature. Hydrolysis, silanol condensation, polymerization, nucleation or aggregation, gelation, aging, and drying were described in detail by Iler and by Brinker and Scherer [5,6,7,8]. These principles remain the mechanistic reference for sustainable silica synthesis, but they must be applied with attention to precursor state. Alkoxide solutions begin from molecular Si(OR)_4_ species, whereas waste-derived water glass liquors may already contain a pH- and history-dependent distribution of molecular, oligomeric, and colloidal silicate/silica. The practical question is therefore not whether the fundamental Si–O–Si network chemistry changes, but how precursor speciation and accompanying ions alter the route and kinetics by which reactive silanol species are generated and polymerized.

### 2.1. The Established Platform in Brief

Alkoxysilanes and alkaline silicate solutions both converge on silanol-rich silicic species that subsequently polymerize into Si–O–Si networks, but they do not begin from equivalent solution states. In the alkoxide route, molecular precursors such as TEOS or TMOS contain hydrolysable Si–OR bonds. Addition of water in the presence of an acid or base catalyst progressively replaces –OR groups with –OH groups, generating partially hydrolysed silanols and, under sufficiently complete hydrolysis, orthosilicic acid, Si(OH)_4_. Condensation between Si–OH groups then produces siloxane bonds, releases water or alcohol depending on the reacting species, and progressively builds oligomers, clusters, and a three-dimensional gel network [5,6,7,8].

The water glass route reaches the same silanol-mediated polymerization stage from a chemically different starting point. Commercial or waste-derived sodium/potassium silicate is an alkaline aqueous silicate system whose composition depends on silica modulus, concentration, pH, temperature, dilution, and production history [43,44]. It may contain deprotonated monomeric silicate, oligomeric species, and a pre-existing colloidal silica fraction. Dilution and acidification do more than trigger precipitation: they promote hydrolysis/protonation equilibria toward Si(OH)_4_ and silanol-rich oligomers, while simultaneously decreasing surface charge and colloidal stability. Consequently, silicic-acid formation, condensation/polymerization, nucleation, aggregation, and colloidal destabilization may overlap rather than occur as discrete sequential events. Water glass should therefore not be represented as a molecular analog of TEOS, even though both routes ultimately polymerize through Si–OH condensation to generate the same Si–O–Si framework.

Drying is a structural operation rather than a finishing step. Ambient-pressure evaporation generates capillary stress that collapses part of the network and yields xerogels with surface areas of roughly 100 to 800 m^2^ g^−1^ and porosities of 20 to 50% [7], whereas supercritical drying removes the liquid–vapor interface and preserves aerogel porosities approaching 90 to 99% at surface areas of approximately 400 to 1200 m^2^ g^−1^ [34,45]. For sustainable systems this single choice carries much of the process cost and much of the environmental burden, and Section 2.2 examines it on that basis.

**Figure 3:**
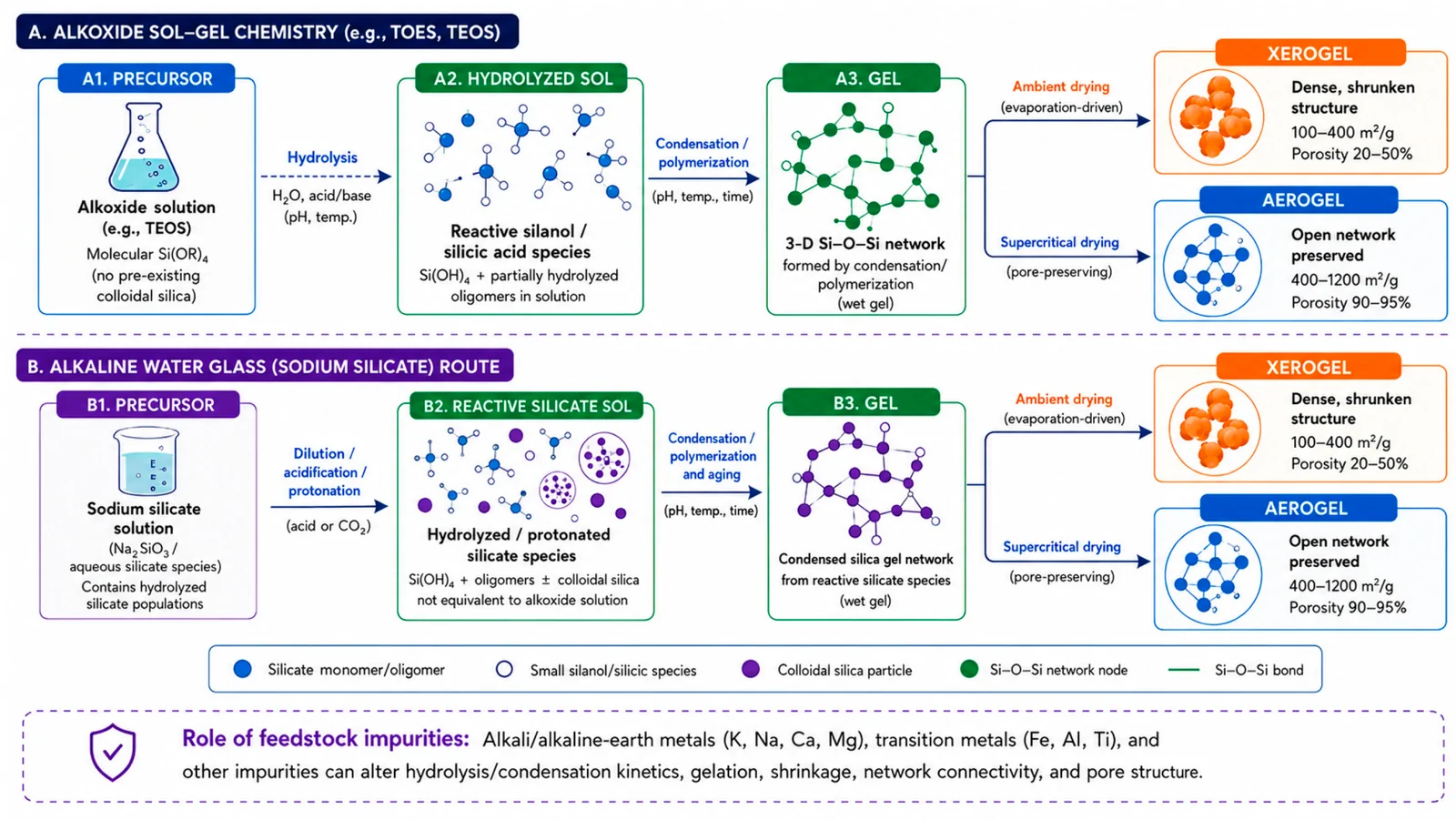
Alkoxide precursors (TEOS, TMOS) begin as discrete Si(OR)_4_ molecules. Hydrolysis produces Si–OH groups and, under full conversion, Si(OH)_4_; these species then condense into a continuous Si–O–Si gel. Alkaline water glass starts differently: an aqueous mixture of molecular, oligomeric, and sometimes colloidal silicates. Acidification and dilution shift the equilibrium toward Si(OH)_4_ and silanol-rich species while destabilizing any colloids, so condensation, aggregation, and network formation overlap. The two routes therefore meet at silanol polymerization but never share the same starting solution. Ambient-pressure drying creates capillary stress and shrinkage; supercritical drying preserves the open pore structure. Residual feedstock ions further influence kinetics, gelation, shrinkage, connectivity, and final architecture.

Figure 3 distinguishes these precursor-specific entry states and their convergence toward reactive Si(OH)_4_/silanol species, subsequent condensation and network formation, and the divergent structural consequences of ambient-pressure and supercritical drying. The figure also highlights the additional role of feedstock-derived ions in modifying reaction kinetics, gelation, shrinkage, network connectivity, and pore development.

### 2.2. Gelation, Aging, Syneresis, and Drying in Waste-Derived Silicate Systems

The gel point is a rheological transition rather than a visual one. It can be defined by the Winter–Chambon criterion, at which the storage and loss moduli exhibit the same power-law frequency dependence and the loss tangent becomes frequency independent [46]. Tilt tests and stopwatch gel times can be useful for screening but do not identify the percolation threshold with the same rigor. For variable alkali silicate extracts, multi-frequency oscillatory rheology therefore provides a defensible basis for comparing gelation across feedstocks, while fast-gelation studies of sodium silicate systems illustrate the strong influence of composition and ionic environment on this transition [47].

Feedstock cations act on the network through identifiable chemical mechanisms. Aluminate substitutes into the silicate framework, forming Si–O–Al linkages that carry a fixed negative charge, lower the solubility of the surrounding silica, and slow the dissolution and reprecipitation that drive aging [6]. Calcium and magnesium bridge deprotonated silanol groups and accelerate aggregation near neutral pH; at higher loadings they precipitate sparingly soluble silicates and consume silicon before the network forms. Sodium and potassium persist as counterions, raise ionic strength, compress the electrical double layer, and shorten gel time by lowering the electrostatic barrier to interparticle contact. Incomplete washing can leave these ions as crystalline salts within the dried pore structure. Iron contributes color and redox activity. Residual carbon presents a heterogeneous nucleation surface and, when it survives into the dried product, contributes microporosity that does not belong to the silica network at all. Each mechanism is individually established, yet the field still lacks systematic measurements of gel time, storage modulus, and Q^n^ distribution as functions of a single impurity concentration in an otherwise fixed silicate system. This is why quantitative impurity–gelation relationships cannot honestly be tabulated at present, and closing that gap should be treated as a research priority rather than passed over as an editorial omission.

Aging continues after the gel point and alters the material more than most reports acknowledge. Dissolution and reprecipitation transfer silica from regions of high positive curvature to the necks between particles, which thickens those necks, stiffens the network, and raises its resistance to capillary collapse. Syneresis, the spontaneous expulsion of pore liquid as condensation proceeds, shrinks the monolith and depends strongly on pH and ionic strength. Silicate solutions recovered from ash age faster than purified equivalents at the same nominal silica concentration because their higher ionic strength promotes both condensation and particle contact, and fast-gelation routes have been developed specifically for sodium silicate systems on that basis [47]. Reported aging protocols for ash-derived gels span a few hours to several days in water, ethanol, or a silicate-containing solution, and the choice of aging medium changes the surface area of the dried product substantially [48]. Aging time, temperature, and medium therefore belong in the methods section of every sustainable silica paper; their omission is one of the more common reasons that published surface areas cannot be reproduced.

Drying decides whether the aged network survives. The capillary pressure developed in a pore of radius r during ambient evaporation follows the Laplace–Young relation, P_c_ = 2γ cos θ/r; so, a 5 nm pore filled with water at a contact angle close to zero experiences stress on the order of tens of megapascals. Networks built from waste silicates are more vulnerable to that stress than alkoxide-derived networks because residual salts and incomplete condensation leave weaker necks. Two routes avoid collapse. Supercritical drying removes the liquid–vapor interface and preserves porosity, but it requires pressure equipment that is difficult to justify economically for a low-cost feedstock. Ambient-pressure drying replaces surface silanol groups with trimethylsilyl or comparable non-condensable groups, usually using trimethylchlorosilane or hexamethyldisilazane after solvent exchange, so that the network springs back once the solvent leaves [49,50]. The second route is the one that has actually been demonstrated on waste feedstocks, and it is where the sustainability argument for waste-derived aerogels stands or falls, because the silylating agent and the exchange solvent frequently dominate both the cost and the environmental burden of the process. Representative textural data for ambient-pressure-dried aerogels prepared from secondary silicate sources are compared in Table 1.

Table 1 shows that waste-derived precursors reach the textural range of alkoxide-derived aerogels, with surface areas of roughly 350 to 500 m^2^ g^−1^. The spread in mean pore diameter, from below 4 nm to above 25 nm, is far wider than that reported for TEOS systems processed identically, and that spread is the practical signature of feedstock variability. It matters for insulation, where pore size relative to the mean free path of air governs the gas-phase contribution to thermal conductivity, and for adsorption, where it governs accessibility. Reporting a surface area without the accompanying pore size distribution therefore conceals the property that feedstock impurities most strongly affect [53].

### 2.3. Feedstock Composition as a Kinetic Variable

pH remains the most consequential of the classical process variables. In alkali silicate systems, its role diverges sharply from that established for alkoxide precursors. Acidification simultaneously modifies silicate charge, speciation, colloidal stability and supersaturation, so that particle morphology is controlled by the complete trajectory of reagent addition rather than by the final pH value. Local acid gradients that arise during scale-up broaden the particle size distribution or trigger premature gelation; consequently, mixing time and addition mode rank as process variables of equal importance to composition itself [7,8,36].

Sustainable feedstocks demand that impurity ions be regarded as kinetic variables, not merely as analytical contaminants. Alkali metals influence silicate solubility and the intensity of subsequent washing; calcium and magnesium form sparingly soluble silicates or alter gelation rates; aluminum introduces aluminosilicate linkages and changes acidity; iron imparts color and redox activity; residual carbon provides heterogeneous nucleation surfaces. These effects explain why identical nominal values of pH, temperature and silicate concentration often produce non-equivalent particles from successive feedstock batches. A mechanistic design strategy therefore requires feedstock-specific response surfaces that relate composition and operating conditions to particle size, Q^n^ connectivity, pore development and yield.

Cross-study comparison becomes feasible only when feedstock and intermediate solutions are characterized using a common minimum dataset. The core set should include feedstock phase and elemental composition by X-ray fluorescence (XRF) and, where trace limits matter, inductively coupled plasma mass spectrometry (ICP-MS); loss on ignition or residual carbon; the SiO_2_/alkali molar ratio and total silica concentration of the extracted liquor; pH and conductivity; and DLS for the colloidal fraction of alkaline water glass intermediates. Process reporting should include acid identity and addition rate, mixing conditions, temperature, gel time, aging medium and duration, wash-water volume and endpoint conductivity, silicon recovery, and drying conditions. Advanced measurements can then be reserved for the optimized feedstock/product pair and the specific application claim.

### 2.4. Classical Architectures as Reference Points

Four architectures serve as the reference points against which sustainable products are usually judged. The Stöber route established that ammonia-catalyzed hydrolysis of an alkoxysilane in alcohol–water mixtures yields monodisperse non-porous spheres of controllable diameter, and it remains the model system for nucleation and growth [25,54]. Surfactant and block copolymer templating produced MCM-41, with ordered channels of tunable width [26,27], and SBA-15, with wider pores, thicker walls, and greater hydrothermal stability [28]. Dendritic silica such as KCC-1 introduced radial fibrous organization that improves surface accessibility and reduces diffusion limitation, and comparable fibrous structures have since been prepared from sustainable precursors [55,56].

Fumed silica provides an important industrial benchmark because it is manufactured continuously by high-temperature flame hydrolysis, yielding non-porous primary particles fused into aggregates and agglomerates rather than discrete sol–gel spheres or ordered mesoporous particles [36]. Its commercial scalability, high purity, rheological activity, and reinforcing performance are exceptional, but the flame route is energy intensive and offers limited intrinsic pore control. Sustainable wet-chemical routes should therefore not be judged solely by whether they reproduce Stöber or mesoporous morphologies; for coatings, rheology, elastomers, and bulk reinforcement, aggregate structure and surface hydroxylation may be more relevant benchmarks. Conversely, pharmaceutical loading and size-selective adsorption generally require the pore accessibility and particle uniformity attainable by sol–gel processing.

Classical silica architectures define what is achievable, but they do not prescribe what a sustainable route must reproduce. The appropriate benchmark is set by the application, and Section 8 orders applications by the tolerance they show to feedstock variability [25,26,27,28,55].

## 3. From Network to Architecture: Structure, Property, and Function

Architecture is the stage at which the chemistry of Section 2 becomes a property. The same hydrolysis, condensation, nucleation, and self-assembly steps generate dense colloids, ordered mesoporous frameworks, hierarchical networks, and hybrid particles, and the architecture selected determines which application the material can serve and how much impurity it can tolerate.

### 3.1. Colloidal, Mesoporous, and Hierarchical Silica

Colloidal silica is the simplest case, comprising dense spheres of narrow size distribution produced by balanced nucleation and growth, and it provides predictable external surfaces but negligible internal loading volume. Template-directed assembly introduces ordered internal porosity: MCM-41 and SBA-15 combine high accessible surface area with size-selective transport, and their pore width, wall thickness, and connectivity respond to template chemistry and synthesis conditions [2,3].

Hierarchical architectures place microporous or mesoporous domains within larger transport channels, which relieves the usual trade-off between surface area and mass transfer and matters wherever rapid transport and extensive surface interaction are required at the same time, as in catalysis and adsorption [2,3].

The same structural principles extend to sustainable silica sources, which can reproduce complex architectures when precursor chemistry and synthesis conditions are appropriately controlled. For example, ordered mesoporous MCM-41 structures have been successfully synthesized from rice husk-derived silica, demonstrating that biomass-derived feedstocks can support the formation of highly organized silica frameworks comparable to those obtained from conventional precursors [57].

Architecture must be selected against the rate-limiting step of the intended application. Dense Stöber particles provide narrow size distributions and reproducible external surfaces but little internal loading volume. Ordered mesoporous structures maximize accessible area and permit pore size matching to small molecules, proteins, or enzymes, yet surfactant removal, pore blocking, and hydrothermal stability become critical. Hierarchical and dendritic structures reduce diffusion resistance but often show broader structural distributions and more complex scale-up. In pharmaceutical systems, larger pores may improve loading of biomacromolecules while also accelerating silica dissolution; narrow pore entrances can suppress crystallization of poorly soluble drugs but may produce incomplete release. Thus, surface area alone is an inadequate descriptor without pore size, pore connectivity, external area, and dissolution behavior.

### 3.2. Core–Shell, Magnetic, and Hybrid Structures

The adaptability of silica chemistry also enables multifunctional architectures in which silica acts as a protective coating, structural framework, or functional support. Core–shell structures represent a prominent example, where a silica layer is deposited around an active core to combine the intrinsic properties of the core material with the chemical stability, surface accessibility, and modification versatility of silica.

Magnetic silica composites, particularly those based on iron oxide cores, illustrate the advantages of this approach. The magnetic component provides external-field responsiveness, whereas the silica shell improves chemical stability, prevents particle aggregation, and introduces functional groups for further modification. These features enable applications in areas such as separation, catalysis, and molecular capture [58,59]. Similarly, silica matrices can serve as supports for metal nanoparticles by providing confined environments that enhance dispersion, reduce sintering, and regulate accessibility to catalytic active sites [60,61].

Hybrid architectures demonstrate that silica functions not merely as a passive structural material but as a versatile platform for integrating multiple functionalities. Importantly, the formation of these systems continues to rely on the same sol–gel principles governing conventional silica synthesis, with additional control introduced through interfacial interactions and composite assembly.

The principal design trade-off in core–shell and hybrid systems is the gain in functionality versus the increase in compositional and regulatory complexity. Each additional core, dopant, fluorophore, metal, polymer, or targeting ligand introduces new interfaces, degradation products, impurity specifications, and analytical requirements. In catalysis these additions may be justified by recoverability or active-site stabilization; in medicine they must produce a clinically meaningful improvement in exposure, targeting, imaging, or therapeutic index. Multifunctionality should therefore be evaluated using a minimum-complexity principle: every component should have a defined function, quantitative benefit, validated stability, and traceable fate.

### 3.3. Structure–Property Relationships

Silica function across different architectures is governed by the combined effects of accessible surface area, pore size and connectivity, and surface chemistry rather than by any single descriptor. Waste-derived precursors add a second layer of control because retained alkali ions, aluminosilicate species, and residual carbon can alter condensation, hydroxylation, and pore accessibility. Structure–property analysis should therefore link these compositional variables to the specific attribute that limits the intended application [2,3].

Structure–property relationships in biological exposure map directly onto pharmacokinetics and toxicology. Particle size, aspect ratio, surface charge, poly(ethylene glycol) (PEG) or zwitterionic surface coverage, and aggregation state govern protein adsorption, macrophage uptake, organ distribution, degradation kinetics, and excretion pathways [62,63,64]. Highly accessible silanol groups can interact with phospholipid membranes and erythrocytes, whereas dense neutral polymer coatings attenuate nonspecific interactions at the cost of slower dissolution. These dependencies underscore the necessity of characterizing the material as administered (including hydrodynamic diameter and colloidal stability in the relevant biological medium) rather than relying solely on dry-state microscopy.

## 4. The Precursor Axis: Sustainable Silicon Sources and Their Chemical Consequences

Section 4 compares sustainable silicon sources according to the chemical state in which silicon is supplied, the accompanying impurities, and the pretreatment required to generate a reactive silicate intermediate. These variables, rather than feedstock origin alone, determine dissolution behavior, solution composition, and the degree of control available during nanoparticle formation.

Five developments, rather than the accumulation of individual feedstock demonstrations, account for the present state of the field. The first is the recognition that controlled combustion below approximately 700 °C preserves biogenic silica in a reactive amorphous form, which makes alkaline dissolution feasible at atmospheric pressure and moderate alkali loading. The second is the substitution of alkaline extraction and controlled neutralization for alkoxide hydrolysis, which removes the petrochemical precursor from the process and shifts the control problem from hydrolysis rate to acidification trajectory. The third is alkali fusion, which converts refractory aluminosilicate residues such as coal fly ash into soluble silicate and thereby opens the largest available waste stream to wet-chemical processing. The fourth is ambient-pressure drying combined with surface silylation, which delivers aerogel-scale porosity without supercritical extraction and is the single change that makes waste-derived aerogels economically plausible. The fifth is the use of already dissolved silicon in fluorosilicic acid and geothermal brine, which removes the dissolution step together with its reagent and energy demand. The sections that follow treat sources and methods separately, then evaluate them against these developments rather than against the number of feedstocks reported.

**Figure 4:**
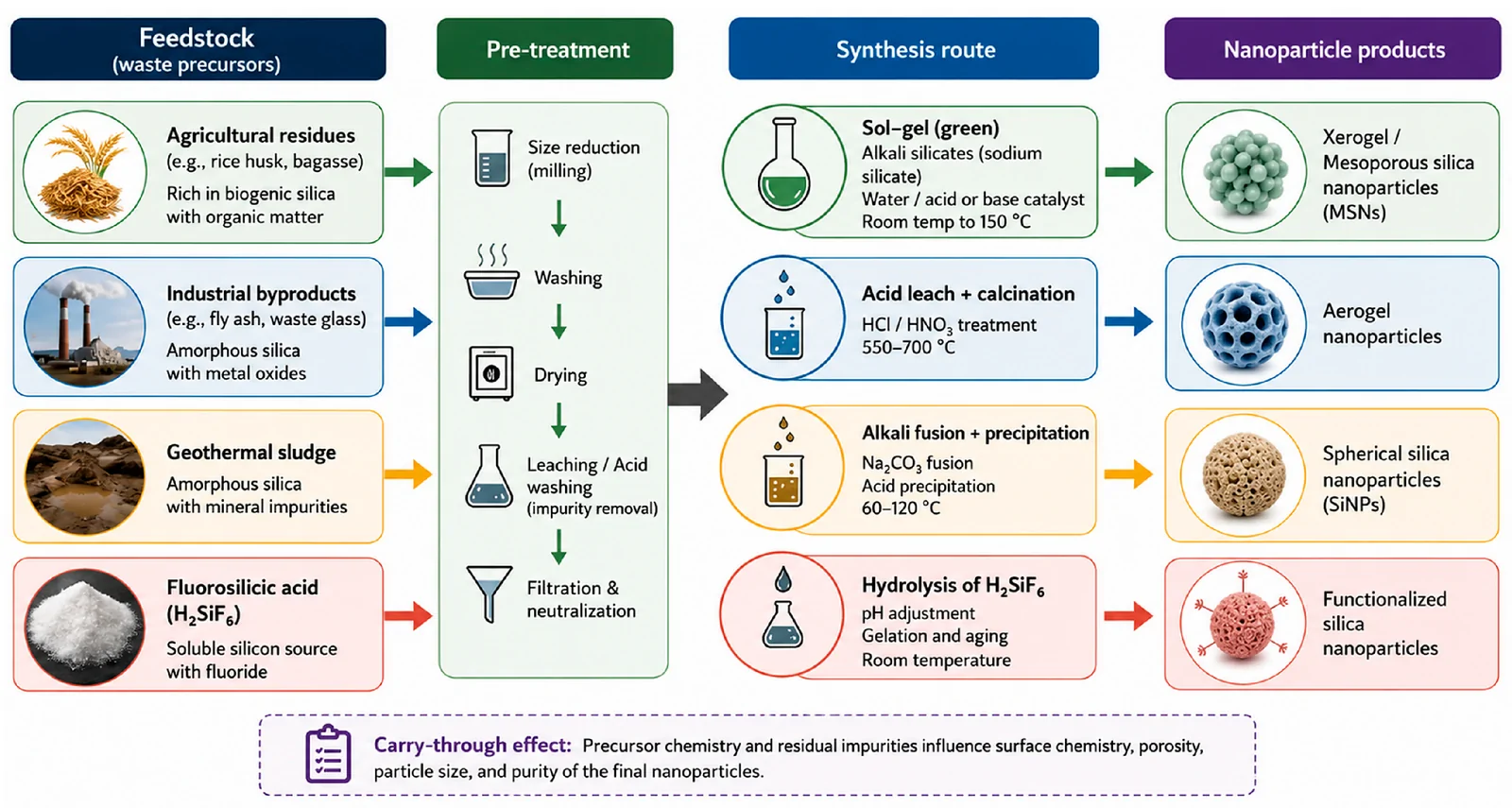
Feedstock-to-nanoparticle conversion pathways. Agricultural residues, industrial by-products, geothermal solids or brines, and fluorosilicate streams require different pretreatment, dissolution, purification, and precipitation strategies. Composition and residual impurities are progressively transferred or removed at each stage and ultimately influence particle size, aggregation, pore architecture, surface hydroxylation, elemental purity, and suitability for environmental, catalytic, agricultural, or biomedical use.

Figure 4 traces how these feedstock attributes are either removed or transferred through pretreatment, dissolution, purification, and precipitation, providing the link between source selection and the final material specification.

### 4.1. Agricultural Residues

Agricultural residues represent the most extensively investigated renewable silica sources because many plants naturally accumulate silicon within their tissues. Among these materials, rice husk ash (RHA) remains the most prominent example due to its high silica content, global availability, and established conversion pathways. Rice husk is a major by-product of rice processing, with global generation exceeding 100 million tonnes annually [18]. The untreated husk typically contains approximately 15–20% SiO_2_ [65,66], which can be concentrated to 85–97% following controlled combustion and ash purification [67,68]. Numerous studies have demonstrated the successful conversion of RHA-derived silica into nanoscale materials through precipitation, sol–gel, and templated synthesis routes [69,70,71,72,73,74,75,76].

The quality of RHA-derived silica is strongly dependent on combustion conditions. Moderate thermal treatment, generally within the range of 400–700 °C, promotes the formation of highly reactive amorphous silica by removing organic components while preserving silicate disorder. In contrast, excessive temperatures induce crystallization into less reactive phases, reducing solubility and complicating subsequent purification and nanoparticle formation [77,78,79,80]. Consequently, combustion history represents a critical processing variable that determines the chemical accessibility of silica.

Mechanistically, biogenic extraction involves four chemically distinct operations: removal or transformation of the lignocellulosic matrix; control of amorphous versus crystalline silica during thermal treatment; dissolution of reactive SiO_2_ into alkali silicate; and re-polymerization during neutralization or sol–gel processing. Acid leaching before combustion can remove K, Ca, Mg, and Fe that otherwise catalyze crystallization or remain in the ash. Alkaline dissolution converts surface Si–O–Si bonds to soluble silicate species, whereas subsequent acid addition decreases silicate charge and drives condensation. Washing then removes counterions and soluble salts. Because each operation changes both yield and reactivity, extraction efficiency should be reported together with silica mass balance, acid/alkali consumption, wash-water demand, and final impurity concentrations rather than only the purity of the recovered powder [77,80,81,82,83,84,85,86,87].

Silicon mass balance deserves closer attention than it currently receives. Acid leaching before combustion removes potassium, calcium and iron efficiently, but it also dissolves part of the silica, and alkaline extraction rarely recovers all of the reactive silica present in the ash; the residue retains crystalline or carbon-encapsulated silica that resists dissolution, and recovery improves measurably when extraction conditions and washing are optimized rather than adopted from an earlier protocol [88]. Combustion atmosphere is a second underreported variable. Oxidative combustion in air removes carbon efficiently but exposes the silica surface to alkali fluxing, whereas pyrolysis under inert gas preserves a carbon–silica composite that needs a separate oxidation step yet suppresses potassium-catalyzed crystallization. Papers that report final purity without silicon recovery, reagent consumption per kilogram of product, and wash-water volume give no basis for judging whether a route is resource efficient.

A major challenge associated with agricultural precursors is compositional variability. Factors including plant variety, soil composition, climate, cultivation practices, and thermal processing conditions can alter silica concentration, mineral composition, and impurity levels [89]. Therefore, reliable production requires standardized characterization of feedstock chemistry and controlled processing strategies to achieve reproducible silica quality.

Sugarcane bagasse ash (SCBA) represents another important silica source derived from agricultural residues due to the scale of sugarcane production and the large quantities of ash generated during biomass combustion. Annual ash production is estimated to exceed 500 million tonnes globally [90]. Raw bagasse contains approximately 14% silica [91], which can increase to 60–89% after combustion and purification [92]. Controlled combustion near 600–700 °C promotes the formation of amorphous silica, with reported silica contents approaching 82% under optimized conditions [93,94]. However, compared with RHA, SCBA generally contains higher concentrations of metallic impurities, including aluminum, iron, calcium, and magnesium, requiring additional purification steps such as acid leaching [95].

Other agricultural residues, including rice straw, corn cob ash, palm oil fuel ash, wheat husk ash, bamboo leaf ash, and barley husk, have also been explored, with reported silica contents ranging from approximately 40 to 96% depending on source and treatment conditions [33,96,97]. Emerging feedstocks, including millet husk, date palm residues, and spent coffee-derived materials, further expand the potential resource base, although their practical implementation remains dependent on regional availability, collection infrastructure, and compositional consistency [12,33].

Agricultural precursors share challenges that extend beyond silica recovery to the control of impurity transfer into the final nanomaterial. Residual potassium, calcium, magnesium, aluminum, and trace metals can modify condensation kinetics, influence particle formation, and alter surface chemistry and pore characteristics [25,33,34,35]. Thus, feedstock characterization and impurity management are fundamental requirements for producing sustainable silica with predictable properties.

Agricultural routes are most compelling when biomass combustion or ash generation already occurs and when processing can be co-located with the residue source. If dedicated high-temperature combustion, long-distance transport, repeated acid washing, and evaporation are required solely to obtain nanosilica, the apparent feedstock advantage may be substantially reduced. The technically superior route is therefore context dependent: rice husk ash may be advantageous for adsorbents, cement additives, or agricultural products at moderate purity, whereas pharmaceutical-grade silica requires tighter trace-metal, microbial, endotoxin, and batch consistency controls that can dominate cost and environmental burden.

### 4.2. Industrial By-Products, Geothermal Deposits, and Fluorosilicic Acid

Industrial by-products combine centralized availability with waste-management value, but their future supply cannot be assumed constant. Coal fly ash (CFA), for example, is abundant in regions that still rely on coal power and typically contains substantial silica together with alumina, iron oxides, calcium phases, and trace metals [98,99]. As coal-fired generation is phased down in many jurisdictions, new CFA production will decline and future silica routes may increasingly depend on legacy ash deposits or alternative residues. Feedstock availability scenarios should therefore be time- and region-specific rather than extrapolated indefinitely from current global generation.

Geothermal silica waste occupies a specialized niche. Dissolved silica in geothermal fluids (about 200 to 1000 mg L^−1^) precipitates as amorphous scale containing roughly 60 to 80% SiO_2_, a structure favorable for nanoparticle synthesis [100,101,102,103]. Its main constraints are co-precipitated arsenic and other trace elements, field-to-field variability, and limited geographic distribution [104]. Fluorosilicic acid (H_2_SiF_6_), a by-product of phosphate fertilizer production at roughly 5 to 6 million tonnes annually, is distinctive because silicon is already dissolved in molecular form, which supports controlled synthesis of mesoporous silica [10,105,106,107,108]. The trade-off is the corrosivity and toxicity of H_2_SiF_6_ and the hydrogen fluoride released on hydrolysis, which demand corrosion-resistant closed systems, fluoride capture, and by-product management.

Three practical separations determine whether these feedstocks are usable. For coal fly ash, magnetic separation removes the iron-rich spinel fraction before alkaline attack, which reduces both coloration and redox activity in the product, and the aluminate remaining in the leachate after silica precipitation can be recovered as a co-product instead of being neutralized as waste. Treating alumina as a co-product rather than an effluent also changes the allocation basis of any subsequent life cycle assessment, which is discussed in Section 9. For geothermal silica, arsenic co-precipitates with the amorphous scale and is not removed by washing alone. Control requires either oxidation of As(III) to As(V) followed by adsorption on ferric phases before silica recovery, or acid redissolution and reprecipitation, and any food-contact or biomedical claim for geothermal silica should be supported by speciated arsenic data rather than a total-element figure. For fluorosilicic acid, hydrolysis and neutralization release hydrogen fluoride, so the process demands closed reactors in fluoropolymer-lined or nickel alloy construction, scrubbing of the vapor space, and conversion of the fluoride to a saleable salt. The fluoride stream, not the silica, governs the environmental performance of that route.

No single sustainable precursor is universally superior. Selection should follow application requirements, regional availability, processing infrastructure, and life cycle performance, and a mature industry will likely draw on a diversified portfolio rather than replacing conventional precursors with one source. Table 2 compares the main feedstocks and shows the underlying trade-off between chemical purity and sustainability.

A useful selection principle is to match impurity tolerance to product function. Residual Al or Ca may be acceptable or even beneficial in construction, adsorption, or catalysis, but trace metals and poorly controlled soluble ions can be unacceptable for optical, electronic, or parenteral pharmaceutical use. Geothermal silica may avoid solid-phase dissolution but requires control of arsenic and field-specific contaminants; fly ash offers scale but demands robust separation of aluminosilicates and toxic metals; and fluorosilicic acid provides molecularly dispersed silicon but transfers the burden to fluoride containment and corrosion-resistant processing. Sustainable sourcing is therefore a constrained optimization problem rather than a universal ranking.

Commercial water glass should also be treated as a chemical feedstock that requires qualification rather than as an impurity-free benchmark. Minor-element content should be measured batch by batch rather than assumed from product grade. Manufacturer grade, SiO_2_/alkali modulus, concentration, and processing history can also change silicate speciation and the colloidal fraction [43,44]. For mechanistic comparisons, supplier specifications should therefore be supplemented by batch elemental analysis and DLS when alkaline silicate solutions are used as starting materials.

### 4.3. Precursor Class as a Determinant of Speciation, Nucleation, and Network Formation

The preceding subsections describe what each feedstock contains. What determines the material outcome is the chemical form in which silicon enters solution and what accompanies it. Four precursor classes can be distinguished on that basis, and this classification predicts synthesis behavior more reliably than a simple waste-versus-virgin distinction. Alkoxysilanes deliver a defined molecular Si(OR)_4_ precursor that must be hydrolysed to Si–OH-bearing species before extensive condensation can occur. Alkali silicates, whether commercial or recovered from ash, enter as alkaline aqueous distributions of silicate species; during dilution and acidification, hydrolysis/protonation equilibria shift toward Si(OH)_4_ and silanol-rich oligomers while any pre-existing colloidal fraction becomes progressively less stable. Condensation/polymerization and aggregation can therefore overlap. Solid amorphous silicates such as ash and geothermal scale must first be dissolved to generate the alkaline silicate intermediate, making dissolution an additional upstream kinetic constraint. Fluorosilicic acid supplies silicon in dissolved molecular form and requires hydrolysis/neutralization together with rigorous fluoride management.

Table 3 summarizes these differences and their consequences. The important distinction is not that alkoxide and water glass routes form fundamentally different silica networks; both ultimately generate silanol-rich species and polymerize through Si–O–Si formation. Their control problems differ because they reach that common chemistry from different initial states. In alkoxide systems, the water-to-silicon ratio, catalyst, and hydrolysis rate govern the buildup of reactive Si–OH species. In alkaline silicate systems, the acidification trajectory, silicate modulus, ionic strength, mixing, and pre-existing colloidal fraction jointly control protonation/hydrolysis, condensation, and aggregation. In solid waste-derived routes, dissolution precedes these solution processes and may itself limit silicon release. Process recipes therefore cannot be transferred between precursor classes without accounting for the step that controls formation and organization of the reactive silanol population.

## 5. The Process Axis: Fundamentals of Sustainable Processing and Emerging Strategies

Changing the silicon source addresses only half of the sustainability problem. The energy, reagent, and solvent demands of a synthesis are set by the processing route, and a waste-derived precursor processed conventionally can carry a larger burden than a purified precursor processed well. This section therefore treats the process as an independent axis, and its organizing question is mechanistic rather than descriptive: which sustainable processing choices act on the kinetics of hydrolysis and condensation, and how do they shift the operating window within which structure can still be controlled?

### 5.1. Conversion Chemistry: Dissolution, Neutralization, Fusion, and Direct Hydrolysis

**Figure 5:**
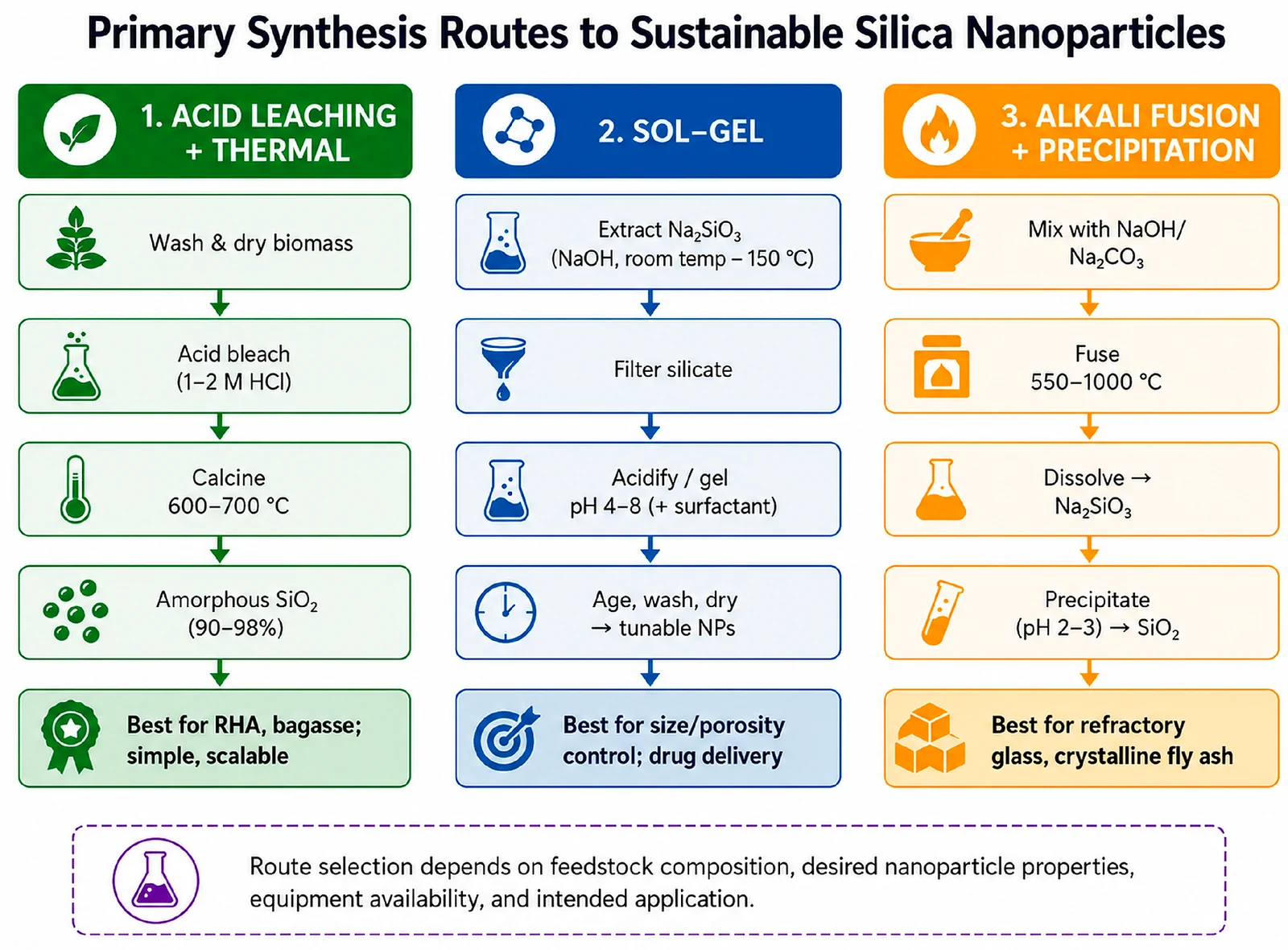
Primary green synthesis routes for sustainable silica nanoparticles. Acid leaching and calcination enrich biogenic silica; alkaline extraction or fusion converts solid silica phases to soluble silicates; controlled neutralization and sol–gel processing regulate nucleation, growth, and gelation; and surfactant or polymer templates generate mesoporous architectures. The preferred route depends on silica speciation, impurity burden, target size and porosity, energy demand, effluent generation, and required product quality.

Once a feedstock is selected, process design must convert its specific silicon phase into a controllable reactive silicate population. The appropriate route therefore follows the precursor state: biogenic amorphous silica is amenable to alkaline extraction; refractory aluminosilicates may require fusion or hydrothermal activation; and dissolved fluorosilicates require hydrolysis/neutralization with fluoride management. Figure 5 summarizes these conversion pathways and the points at which particle or network control is introduced.

Acid leaching followed by calcination remains one of the most widely applied approaches for agricultural residues, particularly rice husk ash. Acid treatment removes metallic contaminants, including potassium, calcium, magnesium, and iron, while thermal treatment eliminates residual organic matter and enriches the silica fraction. This approach provides a relatively simple and scalable route to high-purity silica; however, the resulting materials are often obtained as aggregated powders or micron-scale particles. Additional processing, such as controlled precipitation or sol–gel reconstruction, is therefore frequently required to achieve nanoscale dimensions and tailored architectures.

Sol–gel processing represents a more versatile strategy because it directly exploits the fundamental chemistry governing silica formation. In this approach, recovered silica is commonly converted into soluble silicate intermediates, followed by controlled acidification or condensation to generate nanoparticles or porous networks. Compared with direct thermal approaches, sol–gel routes provide superior control over nucleation, particle growth, pore development, and surface functionality. However, achieving reproducible structures requires careful regulation of parameters such as pH, silicate concentration, catalyst composition, aging conditions, and drying procedure.

Hydrothermal treatment should be distinguished from sol–gel precipitation rather than embedded within it. Depending on feedstock composition and added cations, hydrothermal processing can activate refractory silica, promote dissolution/reprecipitation, or deliberately form secondary silicate and zeolitic phases [109,110]. It is therefore best treated as a feedstock conversion or phase engineering route whose product identity must be verified, not as a generic synonym for water glass sol–gel synthesis.

Manufacturing-scale sol–gel processing combines substantial flexibility with pronounced sensitivity to mixing, residence time distribution, and local concentration gradients. Batch scale-up by geometric enlargement can change the relative timescales of acid addition, micromixing, nucleation, and gelation. Continuous stirred-tank, tubular, segmented-flow, or microreactor approaches can decouple nucleation from growth and improve heat and mass transfer, but they require fouling control and online monitoring. Practical scale-up should therefore identify critical process parameters—silicate concentration, acid-to-silicate ratio, addition rate, mixing energy, temperature, residence time, aging, washing, and drying—and link them to critical quality attributes through design-of-experiments rather than one-factor optimization.

Continuous processing is the element missing from most sustainable silica studies. Batch acidification of an ash-derived silicate is intrinsically non-uniform because the local acid-to-silicate ratio at the point of addition differs from the bulk value, and the discrepancy grows with vessel size. Tubular, segmented-flow and stirred-tank cascade configurations hold that local ratio constant and separate nucleation from growth, which narrows the particle size distribution and makes gel time reproducible. The obstacles are fouling of reactor surfaces by the growing gel and the need to detect the gel point online rather than by sampling. In-line viscometry, ultrasonic time-of-flight measurement, and near-infrared or Raman monitoring of silanol and siloxane bands can each serve as the process analytical measurement, with the gel point as the critical quality attribute to which they are tied. No published study has yet demonstrated continuous production of waste-derived silica gels under online gel point control, and this remains one of the clearest opportunities in the field.

Refractory silica-containing materials, including coal fly ash and mineral residues, can be converted effectively through alkali fusion followed by precipitation. During alkali fusion, silica-containing phases are transformed into soluble silicates at elevated temperatures, typically 450–600 °C, which can subsequently be recovered through controlled precipitation processes [111,112]. This strategy enables the utilization of chemically resistant feedstocks and may allow for simultaneous recovery of other valuable components. Nevertheless, the requirement for high temperatures, substantial alkali consumption, and management of salt-containing effluents remains a limitation for large-scale implementation.

Fluorosilicic acid represents a fundamentally different precursor category because silicon is already available in dissolved molecular form. Controlled hydrolysis and neutralization of H_2_SiF_6_ enable direct formation of high-purity silica nanoparticles and mesoporous materials while avoiding the extensive dissolution steps required for solid feedstocks. Reported approaches have produced silica nanoparticles in the range of 32–85 nm with pore sizes of approximately 2–5 nm through controlled pH adjustment, hydrothermal treatment, and extensive fluoride removal [10,111,112]. However, the corrosive nature of fluorosilicic acid and the potential generation of fluoride-containing intermediates require specialized processing environments and effective waste management strategies.

### 5.2. Low-Energy Processing, Green Reagents, and Biogenic Templating

The processing options considered here are not simply milder versions of established procedures, and treating them as substitutions obscures what they do. Each acts on a specific kinetic term. Microwave irradiation couples directly to the polar solvent and to hydroxylated silicate species, raising the condensation rate at a lower bulk temperature and shortening the interval between nucleation and growth, which narrows the size distribution rather than merely accelerating the reaction. Organic acids used in place of mineral acids buffer the acidification trajectory and slow the approach to the isoelectric region, suppressing the local supersaturation spikes that broaden distributions in scaled batches. Nonionic surfactants and biogenic templates lower the interfacial energy of the forming mesophase and permit ordered porosity at ambient temperature where ionic surfactants require hydrothermal treatment. Ambient-pressure drying does not alter the sol–gel reaction at all; it changes the mechanical problem at the end of it, replacing a pressure vessel with chemical modification of the pore surface. Recognizing which term each option acts on is what separates process design from reagent substitution, and it explains why some green modifications preserve structural control while others degrade it.

Emerging processing routes pursue to reduce energy consumption and chemical inputs while maintaining structural control. Mild room-temperature sol–gel processes using organic acids and nonionic surfactants have demonstrated the formation of mesoporous silica from waste-derived precursors without extensive thermal treatment [113,114,115]. Microwave-assisted synthesis provides accelerated reaction kinetics and improved control over particle uniformity by enhancing nucleation processes [116,117]. Similarly, surfactant-assisted methods employing materials such as cetyltrimethylammonium bromide (CTAB), Triton X-100, and Tween 80 enable improved regulation of pore organization and particle size distribution [118].

Biogenic synthesis strategies represent a further development toward environmentally compatible processing. These approaches employ plant-derived molecules or mild reagents, such as ascorbic acid, to influence silica formation while reducing reliance on harsh chemicals [119,120,121,122]. Although promising, their wider implementation remains dependent on achieving consistent control over reaction mechanisms and demonstrating comparable reproducibility to established sol–gel methods.

Drying carries the largest single share of the energy and capital burden among these options and therefore represents the most consequential intervention point. Supercritical extraction requires a pressure vessel, a solvent inventory, and a cycle time measured in hours, and it scales poorly. Ambient-pressure drying replaces that equipment with solvent exchange and a silylation step, shifting the burden from capital and energy to reagent consumption and solvent recovery. Whether this represents an improvement depends on whether the exchange solvent is recovered and on the choice of silylating agent, since trimethylchlorosilane releases hydrogen chloride while hexamethyldisilazane releases ammonia. Reports that present ambient-pressure drying as intrinsically green without a solvent and reagent balance are therefore incomplete, and the analysis in Section 9 shows that this single decision can determine whether a waste-derived aerogel achieves a lower cradle-to-gate footprint than an alkoxide-derived one.

A comparison of the major synthesis pathways is provided in Table 4, highlighting the inherent trade-offs between structural control, purity, energy requirements, scalability, and processing complexity. Collectively, these approaches demonstrate that sustainable silica synthesis is not defined by precursor origin alone but by the ability to integrate feedstock chemistry with controlled nanostructure formation.

### 5.3. Fundamentals of Process Design: Purity, Yield, and Energy Compared with Conventional Routes

Comparisons of process design should consider silicon recovery, product yield, reagent and water intensity, thermal and electrical duty, and the specification actually achieved. Feedstock price alone is a poor proxy for process efficiency because a nominally free residue can require extensive extraction, neutralization, washing, and drying, whereas a more expensive precursor may reduce downstream operations.

Environmental and economic consequences of these choices are developed quantitatively in Section 9. The process-level principle is that upstream savings from a secondary precursor can be offset by low yield, intensive purification, solvent exchange, or energy-demanding drying [38,39,40,123]. Route selection should therefore minimize burden per unit of product meeting the target specification, rather than burden per unit of raw feedstock.

Waste-derived routes can nevertheless reach conventional ranges of purity, surface area, and structural performance when feedstock variability and impurity transfer are controlled [124,125]. The relevant comparison is therefore performance at matched specification, not precursor origin alone.

Table 2 and Table 4 should consequently be read together: precursor chemistry defines the starting constraints, while process choice determines how much purification, energy, and structural control are needed to meet the application. No route simultaneously maximizes purity, yield, low burden, low cost, and architectural control.

### 5.4. Emerging Strategies at a Glance

The routes described in Section 5.1 and Section 5.2 are sufficiently established for quantitative comparison. A second group remains less mature but is reshaping the direction of the field; consolidating these approaches here clarifies the distinction between demonstrated practice and the current research frontier. Table 5 summarizes eight such strategies, their mechanistic basis, the highest scale at which each has been reported, and the barrier that presently limits it. Three of them, continuous processing, microwave activation, and biogenic templating, were introduced earlier in their process context and are repeated here so that the comparison is complete; mechanochemical activation builds on the mechanical treatment of fly ash already established for cementitious use [35].

Table 5 reveals two informative patterns. The first is that the strategies closest to deployment act on transport and mixing rather than on chemistry. Continuous processing and mechanochemical activation change how reagents meet and how quickly a solid dissolves, and both can be engineered with existing equipment. The strategies furthest from deployment act on the chemistry itself, and each is limited by a materials problem rather than by process design: enzyme cost and stability in the biomimetic case, solvent viscosity and product recovery in the eutectic-solvent case. The second pattern is that readiness is inversely related to the elegance of the concept. Enzyme-mediated silicification reproduces at neutral pH and ambient temperature what industrial routes achieve with strong base or high temperature, and the discovery that sponge silicateins and diatom silaffins catalyze siloxane condensation under physiological conditions remains the most instructive result in this table [126,127,128]. It is also the least likely to reach production in its present form, and its practical value probably lies in synthetic polyamine analogs that reproduce the catalytic function at reagent cost.

One absence from Table 5 deserves explicit statement. None of these strategies has been demonstrated on a waste-derived silicate under conditions that would allow its energy and reagent balance to be compared with the conventional route it is intended to replace. Biomimetic and enzymatic silicification has been studied almost entirely with purified alkoxysilanes or silicic acid solutions, and deep eutectic solvents have been applied to silica mainly as a post-synthesis functionalization medium rather than as a synthesis medium [129]. Until these strategies are tested against a real ash-derived silicate, with its alkali load and its batch-to-batch variability, their reported advantages remain provisional. Deciding which of them to pursue is an empirical question, and the field has not yet designed the experiments that would answer it. Plasma-based routes are omitted from this comparison for a related reason: high-temperature plasma is already the basis of the energy-intensive fumed-silica process, and non-thermal plasma has not been shown to produce silica from a silicate solution at useful yield; so, listing it as an emerging sustainable strategy would misrepresent the evidence.

## 6. Characterization, Quality Attributes, and Structure Verification

Characterization of sustainable silica should be rigorous but staged. During process optimization, a restricted core set can provide most of the information needed to compare conditions: feedstock composition and silica phase, silica concentration/modulus of the extracted liquor, pH and conductivity, silicon recovery, DLS or an appropriate particle size measurement, XRD/FTIR confirmation, and BET analysis when porosity is a target. Once a route is optimized, the selected feedstock and product should undergo the deeper structural, surface, impurity, and application-specific analyses needed to support the scientific claim. This tiered strategy reduces unnecessary analytical burden for laboratories with limited instrument access without lowering the evidence standard for the final material.

The analytical depth should therefore be proportional to the claim. A process development paper may be publishable with a validated core dataset if it demonstrates reproducible optimization and mass balance, whereas claims of mechanistic control, regulated use, or advanced architecture require orthogonal methods and tighter impurity evidence. Early patent filing can legitimately precede complete product qualification, but intellectual-property timing does not substitute for the characterization needed to support a later scientific publication. This distinction separates practical process development from full application qualification.

### 6.1. Physicochemical and Advanced Characterization Techniques

X-ray diffraction (XRD) provides the first assessment of silica structural organization by distinguishing amorphous silica from crystalline phases. A broad diffraction halo is characteristic of the disordered siloxane network associated with reactive amorphous silica, whereas sharp reflections corresponding to quartz or cristobalite indicate undesirable crystallization, often resulting from excessive thermal treatment during biomass combustion. Because amorphous silica exhibits higher chemical reactivity and solubility than crystalline forms, XRD analysis is particularly important for evaluating the effectiveness of thermal pretreatment strategies.

Fourier-transform infrared spectroscopy (FTIR) provides rapid information on siloxane bonding, surface hydroxylation, and residual organics. Raman spectroscopy can provide complementary network information. Both methods can assist in tracking changes associated with Q^n^ connectivity and are substantially faster than solid-state ^29^Si NMR, but overlapping bands and matrix effects limit quantitative assignment; ^29^Si NMR remains the more direct method for resolving Q^2^/Q^3^/Q^4^ populations when network connectivity is central to the claim.

Nitrogen adsorption–desorption analysis using Brunauer–Emmett–Teller (BET) and Barrett–Joyner–Halenda (BJH) methods is essential for evaluating porous structure. These measurements provide specific surface area, pore volume, and pore size distribution, which are among the most important descriptors governing silica performance. Sustainable silica materials commonly exhibit Type IV adsorption isotherms associated with mesoporous structures. The nature of the hysteresis loop provides additional information on pore geometry: H1-type hysteresis generally indicates relatively uniform open pores, whereas H2-type hysteresis suggests narrower pore necks and more complex pore connectivity [130].

Electron microscopy techniques, including scanning electron microscopy (SEM) and transmission electron microscopy (TEM), provide direct visualization of particle morphology, size distribution, aggregation behavior, and, where applicable, ordered pore arrangements. These observations are particularly important for sustainable silica because variations in precursor composition and impurity content can influence nucleation and growth processes, leading to broader particle size distributions than those commonly obtained from purified alkoxide precursors.

Thermogravimetric analysis (TGA) evaluates the thermal stability and composition of silica materials by quantifying adsorbed water, residual organic species, and carbonaceous impurities. This information is especially relevant for biomass-derived silica because incomplete removal of organic components can affect purity, surface chemistry, and subsequent material performance.

Surface charge and colloidal stability are commonly evaluated through zeta-potential measurements, which provide information on particle–particle interactions and suspension behavior. In addition, solid-state silicon nuclear magnetic resonance spectroscopy (^29^Si NMR) provides molecular-level insight into silica network condensation. The relative abundance of Q^2^, Q^3^, and Q^4^ silicon environments reflects the degree of siloxane connectivity and surface silanol density. Compared with highly condensed alkoxide-derived silica, waste-derived materials frequently exhibit higher proportions of Q^2^ and Q^3^ species, indicating increased hydroxylation, structural defects, and incomplete condensation.

The combined use of these techniques establishes the relationship between synthesis conditions, silica structure, and resulting material properties. No single analytical method provides a complete description of sustainable silica; rather, a combination of structural, chemical, and surface analyses is required to verify reproducibility and application suitability.

Characterization is best interpreted as a linked evidence chain rather than a checklist. XRD establishes phase state; FTIR and Raman probe bonding; BET/BJH measurements characterize accessible porosity; microscopy samples morphology; DLS is intensity weighted and sensitive to aggregates [37]; and zeta potential depends on medium composition. For alkaline water glass sols, DLS should be included explicitly [43,44], while ultrasonic attenuation or related acoustic techniques can be valuable for concentrated or optically opaque sols where multiple light scattering limits conventional DLS. Elemental analysis by ICP-OES/ICP-MS should include color-forming ions such as Fe, Mn, and Cr when optical appearance or coating use is relevant. Helium pycnometry can provide true density and help distinguish dense silica from residual carbon or secondary mineral phases. X-ray photoelectron spectroscopy (XPS) can resolve near-surface composition, but insulating silica powders require careful mounting, charge compensation, calibration, and interpretation; XPS should therefore complement rather than replace bulk elemental analysis. Solid-state ^29^Si NMR remains the reference technique for Q^n^ populations, with FTIR/Raman serving as faster but less quantitative screening methods.

### 6.2. Assessing Sustainability Through Material Characterization

Sustainability-oriented characterization should determine whether a processing step improves a material attribute enough to justify the resources consumed. For purification, combustion, extraction, washing, or drying, measurements should therefore be paired across the process: feedstock or intermediate composition, silicon recovery, residual impurities, phase state, and the critical product attribute targeted by that step. This approach distinguishes genuine quality improvement from burden shifting.

The interpretation is application-dependent. Removing an impurity is beneficial only when its residual level affects function, safety, or reproducibility; likewise, more severe thermal or washing treatment is not an improvement if it lowers silicon yield or damages the desired amorphous or porous structure. Characterization thus provides the evidence needed to set a fit-for-purpose purification endpoint rather than defaulting to maximum purity.

The required analytical depth should be application specific but predefined. A construction additive may be controlled by phase, composition, moisture, and particle size distribution; an adsorbent additionally requires accessible area, pore structure, and leaching behavior; a catalyst support requires acidity, thermal stability, and metal–support interactions; and a pharmaceutical carrier requires a far broader set of critical quality attributes, including size distribution, aggregation, pore loading, surface-ligand density, residual surfactant, elemental impurities, endotoxin, sterilization compatibility, dissolution, and biological identity. Establishing acceptance ranges for these attributes is a prerequisite for meaningful batch comparability and scale-up.

### 6.3. Rheological and Network-Level Characterization of Silica Sols and Gels

The techniques described above establish what a dried silica powder is. They say little about how the network formed, and for sustainable feedstocks that omission matters, because the impurity effects set out in Section 2.2 act during gelation and aging rather than after drying. Three families of measurement close the gap. Small-amplitude oscillatory rheology defines the gel point through the frequency independence of the loss tangent and quantifies network stiffness through the storage modulus [46]; a time sweep at a single frequency records the induction period, and a frequency sweep at fixed conversion identifies the critical gel. Solid-state ^29^Si magic-angle-spinning NMR resolves the Q^2^, Q^3^ and Q^4^ environments and gives the degree of condensation directly, which is the parameter that separates a well-aged network from an incompletely condensed one of identical surface area. Running ^27^Al MAS NMR on the same samples distinguishes framework aluminum in tetrahedral coordination from extra-framework octahedral species, and therefore shows whether feedstock aluminum has entered the network or is merely occluded. Small-angle X-ray and neutron scattering give the fractal dimension and correlation length of the wet gel, neither of which can be recovered from nitrogen adsorption on the dried solid.

Two further methods deserve routine use. Mercury intrusion porosimetry covers the macropore range that nitrogen adsorption cannot reach and is necessary for monolithic gels and granular adsorbents. Comparing nitrogen uptake with the uptake of a larger probe molecule separates total porosity from accessible porosity, a distinction that becomes important whenever residual carbon or occluded salt blocks part of the pore network. A practical minimum for a sustainable silica gel study is therefore a rheological gel point, a ^29^Si degree of condensation, a full nitrogen isotherm with its pore size distribution rather than a single surface area value, elemental analysis by ICP-MS including the regulated elements, and thermogravimetric determination of residual carbon and water. Reporting these five together would permit the direct feedstock-to-feedstock comparison that the current literature does not support.

## 7. Surface Functionalization and Interfacial Reactivity

The surface chemistry of silica represents a defining feature that distinguishes it from many other inorganic nanomaterials. The abundance of surface silanol (Si–OH) groups generated during silica formation provides a versatile platform for chemical modification, enabling precise control over interfacial interactions, dispersion behavior, and functional performance. These surface hydroxyl groups serve as reactive sites for introducing organic functionalities, anchoring molecules, and tailoring interactions with surrounding environments. Consequently, surface functionalization transforms silica from a passive structural material into an application-specific platform with controlled chemical reactivity.

The density, distribution, and accessibility of silanol groups are strongly influenced by synthesis conditions, network condensation, and thermal or drying history. Their local configuration also matters: isolated, vicinal, and geminal silanols differ in hydrogen bonding, acidity, hydration, and accessibility to grafting reagents. Sustainable silica may shift the relative abundance of these sites through residual ions and incomplete condensation, so total hydroxyl content alone is insufficient when surface reactivity is central to the application. Where feasible, spectroscopic or probe-based measurements should therefore distinguish silanol population and accessibility rather than reporting only nominal functionalization conditions.

### 7.1. Common Strategies

Silanization remains the most established strategy for modifying silica surfaces because it provides a versatile route for converting surface silanol groups into chemically tailored interfaces while preserving the underlying silica framework. The process typically involves condensation between organosilane molecules and surface hydroxyl groups, generating stable Si–O–Si bonds and introducing organic functionalities with controlled chemical characteristics. The broad versatility of silane chemistry enables the incorporation of diverse functional groups, including amines, thiols, carboxylic acids, phosphonates, epoxides, and chelating ligands, allowing silica surfaces to be engineered for selective interactions with inorganic ions, organic molecules, catalysts, and biological species [58,59,131,132].

Amino functionalization with (3-aminopropyl)triethoxysilane (APTES) is one of the most widely investigated modification strategies because the introduced primary amine groups provide strong coordination sites for metal ions, enhance interactions with negatively charged molecules, and offer versatile platforms for subsequent coupling reactions. APTES-modified silica has been extensively applied in adsorption, separation, biosensing, and catalytic systems because amine groups can simultaneously modify surface charge, binding affinity, and chemical accessibility [131,132]. Similarly, thiol-functionalized silica exhibits strong affinity toward soft metal ions through sulfur coordination, whereas carboxyl-containing surfaces provide additional binding mechanisms through electrostatic interactions, hydrogen bonding, and metal–carboxylate complexation.

Molecular recognition ligands extend silica functionalization beyond simple surface groups and enable highly selective interfaces. Chelating molecules such as phenanthroline derivatives introduce specific coordination environments that enhance affinity toward transition metals compared with unmodified silica. Recent studies using phenanthroline-functionalized silica systems have demonstrated that ligand immobilization within porous silica frameworks can generate selective sorbents for trace metal extraction by combining the high surface area of silica with the strong complexation ability of organic ligands [133,134,135]. In particular, AlSuhaimi and co-workers reported porous silica monoliths functionalized with 5-amino-1,10-phenanthroline for selective solid-phase extraction of transition metals, demonstrating the potential of ligand-engineered silica surfaces for analytical separation applications. More recently, phenanthroline-2-carbaldehyde-functionalized mesoporous silica nanoparticles prepared using a sodium silicate-derived silica source showed efficient chelation and extraction of trace metals from wastewater matrices, illustrating how surface modification can extend sustainable silica platforms toward selective environmental applications.

Other ligand-based approaches further demonstrate the adaptability of silica surface chemistry. Bisphenol A-functionalized mesoporous silica nanoparticles have been developed to introduce selective interactions toward organic contaminants, while aryl diazonium-mediated modification provides an alternative covalent functionalization pathway for constructing chemically robust silica interfaces. These examples highlight that silica functionalization is not limited to increasing adsorption capacity but enables deliberate molecular design of surface environments with specific recognition properties.

Functionalization can generally be achieved through two complementary strategies: co-condensation and post-synthetic grafting. In co-condensation, functionalized silane precursors are introduced during silica network formation, resulting in the incorporation of organic groups throughout the developing framework. This approach can provide relatively uniform functional group distribution and improved incorporation efficiency but may influence pore formation, network organization, and accessibility of the functional sites. In contrast, post-synthetic grafting modifies preformed silica surfaces after particle or pore formation, offering greater control over surface coverage while generally preserving the original structural characteristics and pore accessibility [26,27,58].

The selection between co-condensation and grafting depends on the intended balance between functional group density, accessibility, stability, and structural preservation. Co-condensation is advantageous when homogeneous distribution of functionalities throughout the silica matrix is required, whereas post-synthetic grafting is preferred when accessible surface modification and the preservation of pre-existing pore architecture are critical. Therefore, functionalization strategy represents an additional structural design parameter, similar to particle size and porosity, that determines the final performance of silica-based materials.

Drug delivery and diagnostic systems require control of functional group location and density as well as confirmation of their nominal presence. Co-condensation can place groups within pore walls and internal surfaces, potentially reducing accessibility or altering condensation and dissolution. Post-synthetic grafting favors accessible external and pore surface modification but can produce gradients and pore mouth blockage. Quantification should therefore combine elemental analysis, thermogravimetry, spectroscopy, and, where possible, ligand-specific assays. Reporting only the amount of silane added is insufficient because grafting efficiency depends on surface hydration, silanol type, solvent, water activity, temperature, and pre-existing impurities.

### 7.2. Impact on Adsorption, Catalysis, and Stability

Surface functionalization directly controls the interactions between silica and target molecules, making it a decisive factor in adsorption, catalysis, separation, and colloidal applications. In adsorption systems, the introduction of specific binding groups enhances interactions beyond the nonspecific adsorption provided by bare silica. Amine-, thiol-, and chelating ligand-functionalized surfaces exhibit improved affinity and selectivity toward metal ions and organic molecules by providing tailored coordination environments [131,132]. The combination of accessible pore networks with chemically active surfaces enables silica materials to achieve selective capture while maintaining high adsorption capacity.

Catalytic systems use silica both as a structural support and as a chemically tunable interface. Surface functional groups can immobilize catalytic species through coordination or covalent interactions, improving dispersion and reducing nanoparticle aggregation. In mesoporous silica systems, the combination of ordered pore architecture and controlled surface chemistry facilitates uniform distribution of active components while maintaining accessibility to reactants [58,59]. Thus, catalytic performance emerges from the combined influence of pore accessibility, surface interactions, and active-phase stabilization.

Surface modification also plays a central role in controlling the behavior of silica nanoparticles in suspension. At the nanoscale, particle stability is governed by the balance between attractive forces that promote aggregation and repulsive interactions that maintain dispersion. Functional groups introduced onto silica surfaces modify electrostatic charge, hydration behavior, and steric interactions, thereby influencing colloidal stability and transport properties.

Biological fluids rapidly transform the engineered silica surface into a dynamic interface of adsorbed proteins, lipids, and metabolites. Surface charge, hydrophobicity, curvature, and ligand density shape this corona and thereby alter receptor recognition, cellular uptake, complement activation, and circulation. PEGylation, defined here as surface grafting with poly(ethylene glycol) (PEG), or zwitterionic coating can reduce nonspecific adsorption and aggregation, but dense coatings may also shield targeting ligands and retard silica hydration and dissolution. Surface engineering should therefore be assessed in the intended medium and exposure route rather than inferred from measurements in water alone.

Surface engineering can additionally regulate the physical durability of porous silica materials. Hydrophobic modification of surface silanols provides a notable example, where replacing hydrophilic hydroxyl groups with nonpolar functionalities reduces water affinity and minimizes capillary stress during drying. This strategy enables improved preservation of highly porous silica structures, including aerogels, under less demanding processing conditions [136]. Therefore, surface chemistry influences both molecular-level interactions and macroscopic structural stability.

### 7.3. Green Functionalization Approaches

The sustainability profile of silica nanomaterials is determined not only by the origin of the silica precursor but also by the chemistry used to engineer its surface. Conventional functionalization strategies frequently rely on organic solvents, excess silane reagents, prolonged reaction times, and additional purification steps, which may reduce the environmental benefits achieved through sustainable silica production. Therefore, the development of greener surface modification approaches represents an essential extension of sustainable silica design, aiming to maintain functional performance while reducing chemical consumption, energy input, and waste generation.

Several strategies have been explored to improve the environmental compatibility of silica functionalization, including aqueous-phase grafting, solvent-minimized protocols, room-temperature modification, and the use of renewable or biobased modifiers. These approaches seek to replace conventional organic-solvent-intensive procedures with milder reaction conditions while preserving efficient attachment of functional molecules to silica surfaces. Recent reviews have highlighted that greener silica technologies increasingly focus on reducing hazardous reagents, lowering energy requirements, and minimizing waste throughout both synthesis and post-synthetic modification stages.

The selection of functionalization chemistry is particularly important because surface modification can represent a significant fraction of the environmental burden associated with the final nanomaterial. Approaches based on water-compatible silanes, biodegradable modifiers, and multifunctional molecules capable of achieving high surface coverage at lower reagent concentrations offer promising pathways toward more sustainable silica interfaces. However, sustainability must be evaluated based on the entire modification process, including reagent synthesis, solvent recovery, purification requirements, and life cycle impacts rather than solely on the renewable origin of the modifier.

Waste-derived silica may provide additional opportunities for reducing functionalization intensity because these materials often contain a greater proportion of accessible surface hydroxyl groups compared with highly condensed silica prepared from purified alkoxysilanes. Increased silanol availability can enhance surface reactivity and improve grafting efficiency, potentially reducing the quantity of functionalizing agents required to achieve the desired surface properties. Nevertheless, the heterogeneity of many waste-derived materials requires careful control because residual inorganic species and structural defects may influence grafting uniformity and surface accessibility.

Sustainable functionalization therefore requires the integration of surface chemistry with green processing principles. The objective is not simply to introduce additional functionality but to design silica interfaces in which chemical performance, resource efficiency, and environmental compatibility are optimized simultaneously. Such an approach is essential for translating sustainable silica nanoparticles from laboratory-scale materials into reproducible platforms for advanced technological applications.

A credible green functionalization claim should include solvent hazard, reagent atom efficiency, excess silane, reaction concentration, temperature, purification, solvent recovery, and functional performance per unit mass of product. Aqueous grafting is not automatically superior if it causes uncontrolled oligomerization and repeated washing, and a bio-derived ligand is not necessarily low impact if its isolation is resource-intensive. The appropriate objective is reduced life cycle burden at an equivalent functional specification, supported by mass balance and performance-normalized comparison.

## 8. Applications Ordered by Tolerance to Feedstock Variability

Section 8 uses representative application fields, rather than cataloging every reported use of silica nanoparticles, to demonstrate how the fundamental variables discussed in the preceding sections—precursor chemistry, network connectivity, particle size, pore architecture, surface silanol density, functionalization, and impurity control—translate into function. Environmental remediation, catalysis, agriculture, energy and sensing, coatings and composites, and biomedical systems are therefore treated as equally informative examples of the versatility of nanostructured silica. Together, they show how materials derived from sustainable feedstocks can enter both high-volume and high-value markets when their structural characteristics are matched to application-specific requirements.

Sustainable precursor origin does not, by itself, guarantee superior performance or lower environmental burden. Waste-derived silica is technologically meaningful only when it achieves the required purity, reproducibility, morphology, porosity, surface chemistry, stability, and service life with a demonstrable reduction in resource or energy demand. The examples below are consequently interpreted through a common structure–function framework rather than ranked by application importance. Table 6 summarizes selected systems and the principal evidence required to move from proof-of-concept performance toward reliable use.

The applications below are ordered by how much of the performance derives from the silica network rather than from the particle surface alone. Aerogel insulation, monolithic and granular adsorbents, chromatographic packings, and controlled-release matrices depend on a continuous pore network that is fixed during gelation and aging, so feedstock effects propagate into them directly; silica aerogel microparticles prepared from rice husk ash and loaded with a poorly soluble drug illustrate how far this can already be taken with a waste feedstock [137]. Reinforcing fillers, anti-caking agents and coating additives depend mainly on aggregate structure and surface hydroxylation, and they tolerate feedstock variability far better. That ordering is also the practical entry sequence for waste-derived silica: the applications with the widest impurity tolerance can absorb feedstock variability today, whereas the network-controlled applications require the process control described in Section 2.2 and Section 6.3 before they become dependable.

### 8.1. Environmental Remediation and Molecular Separation

Environmental adsorption is one of the clearest demonstrations of the relationship between silica architecture and function. A highly accessible surface area provides a large population of adsorption sites, mesopores facilitate the transport of dissolved species, and surface silanol groups permit the attachment of amines, thiols, chelating ligands, and hydrophobic functionalities. The resulting materials can be designed for metal-ion capture, dye and pharmaceutical removal, preconcentration, or selective separation, depending on pore dimensions and the chemistry of the target–surface interaction [15,109,110,131,132].

Waste-derived silica systems illustrate this potential across chemically different contaminants. Fly ash-derived floral nanosilica has been reported to remove approximately 90–95% of Cd^2+^ and 85–92% of chromium species under study-specific conditions, whereas rice husk-derived and sodium silicate-derived mesoporous materials have been used for dyes, bisphenol A, amoxicillin, and related organic contaminants [115,134,138,139]. These examples show that sustainable silica can supply both the porous scaffold and the reactive surface required for molecular capture; however, performance depends strongly on pH, ionic strength, competing species, particle aggregation, and the accessibility of grafted groups.

The chemical complexity of sustainable feedstocks can be either beneficial or detrimental. Residual aluminum or metal species may create additional Lewis-acidic or ion-exchange sites, but uncontrolled impurities can block pores, leach into treated water, or interfere with selectivity. Accordingly, the purification target should be determined by the intended separation rather than by a universal maximum-purity criterion. Bulk water treatment media may tolerate defined minor phases that would be unacceptable in analytical preconcentration or biomedical purification, provided that leaching and secondary contamination are excluded.

High-impact evidence in this field requires more than percentage removal from a single-solute model solution. Adsorption capacity should be normalized to sorbent mass and, where informative, accessible surface area; equilibrium and kinetic models should be physically justified; realistic matrices and competing ions should be tested; and regeneration, contaminant recovery, sorbent attrition, and end-of-life fate should be quantified. From an investment perspective, the strongest opportunities are likely to arise where locally abundant residues can be converted near the waste source into regenerable adsorbents or separation media, thereby combining disposal savings with a clearly defined treatment function.

### 8.2. Catalysis and Chemical Processing

Silica is widely used in catalysis because its high-area framework can disperse active metals, enzymes, organocatalysts, or acidic and basic functionalities while maintaining access to reactants. Mesopore diameter and connectivity regulate mass transport; silanol density and grafted ligands control anchoring strength; and wall thickness influences hydrothermal and mechanical stability. Thus, catalytic performance emerges from the combined effects of active-phase chemistry, interfacial bonding, pore transport, and support durability rather than from surface area alone [58,59,60,61,140,141].

Porous silica obtained from rice husk, fly ash, and related residues has been investigated as a support for palladium and other catalytic phases. High coupling yields and catalyst reuse have been reported for Suzuki–Miyaura and related transformations, indicating that sustainable silica can provide an effective dispersion medium for high-value catalytic applications [140,141]. The economic attraction is not simply lower precursor cost: converting a residue into a reusable support can also reduce waste management burdens and enable recovery of expensive active metals at the end of catalyst life.

Mechanistic comparison with conventional supports must nevertheless isolate the effect of silica origin from differences in active metal loading, particle size, dispersion, oxidation state, support acidity, and reaction conditions. Residual heteroatoms may generate useful acid sites or modify metal–support interactions, but they may also promote side reactions, accelerate deactivation, or complicate product purification. Matched benchmarking should therefore include active-phase loading, accessible surface area, turnover metrics, selectivity, leaching, pore stability, and performance after repeated cycles.

Industrial implementation requires catalyst support synthesis to be integrated with washing, drying, shaping, and regeneration. Continuous precipitation or sol–gel processing, controlled incorporation of heteroatoms, and recovery of active metals from spent catalysts could create higher-value outlets for sustainable silica than its use as an undifferentiated filler. Investment decisions should be based on catalyst productivity, service life, regeneration efficiency, and avoided disposal cost, rather than on the nominal price of silica alone.

### 8.3. Agriculture and Controlled Agrochemical Delivery

Agriculture provides a particularly direct circular economy pathway because silica-rich crop residues can be converted into materials that return functional silicon to agricultural systems. Silicon can strengthen plant tissues, influence water relations, support antioxidant responses, and mitigate selected abiotic stresses. At the nanoscale, high dispersibility and reactive surface area may increase contact with plant or soil environments, while porous silica can act as a carrier for nutrients or crop protection agents [142,143,144,145,146,147,148,149].

Rice husk ash can retain potassium and therefore offers potential fertilizer value; it has also been used as a feedstock for silicon–potassium slow-release fertilizer [150]. This advantage should not be generalized to purified nanosilica, because acid leaching and washing may remove much of the soluble K. Agricultural claims should therefore report potassium content, release behavior, and contaminant limits in the final formulation rather than infer fertilizer value from the starting ash.

Rice husk-derived SiNPs and related agricultural silica materials have been reported to improve growth or stress tolerance under salinity, drought, and metal exposure. Yield increases of approximately 10–25% and reduced sodium accumulation have been described at concentrations commonly within 50–200 mg L^−1^, although responses depend on crop species, particle characteristics, dose, exposure route, and experimental conditions [142,143,145,146,151]. These findings demonstrate application potential but do not yet define universal agronomic recommendations.

Porous nanosilica carriers extend this concept beyond direct silicon supplementation. Pore volume, surface charge, and functional coatings can be adjusted to regulate nutrient, pesticide, or biostimulant loading and release. The same architecture–transport principles used in adsorption and drug delivery therefore apply to agrochemical systems, but the relevant media are soil solution, leaf surfaces, root exudates, and irrigation water. Release must be synchronized with biological demand while avoiding rapid wash-off, persistent residues, or non-target exposure [148,149].

Translation requires multi-season field trials, dose–response analysis, comparison with conventional silicon fertilizers or formulations, and measurement of soil transformation, runoff, microbial effects, and crop residues. Feedstock-derived metals and soluble salts require particular attention because agricultural applications may involve repeated and geographically broad exposure. Commercial opportunity is strongest where production can be co-located with rice, sugar, or biomass-processing facilities, allowing regional residues to support locally adapted fertilizers, carriers, or soil amendments with verifiable agronomic benefit.

### 8.4. Energy, Sensing, and Analytical Platforms

Silica nanostructures contribute to energy technologies as porous templates, interfacial coatings, catalyst supports, separators, and precursors for silicon-containing electrode materials. Ordered or hierarchical pores can facilitate ion transport and confine electroactive phases, whereas silica shells can suppress aggregation and stabilize interfaces during cycling. In electrocatalysis and energy storage, these functions depend on pore accessibility, wall stability, electrical integration with conductive components, and the controlled conversion or retention of the silica framework [3].

The same combination of high area and tailorable surface chemistry supports sensing and analytical applications. Functionalized silica can preconcentrate target molecules, immobilize recognition ligands, protect optical or electrochemical reporters, and regulate diffusion to a transducer. Phenanthroline-, amine-, and other ligand-modified silica systems illustrate how molecular recognition can be coupled to porous transport and selective extraction [131,132,133,134,135,152]. For sensors, reproducibility of ligand density, particle dispersion, signal stability, response time, interference tolerance, and regeneration are more important than maximizing surface area in isolation.

Energy, sensing, and analytical applications offer potentially high-value outlets for sustainable silica but also impose demanding specifications. Trace metals may alter electrochemical stability or baseline signals, while residual carbon can be either a useful conductive component or an uncontrolled source of variability. Sustainable routes should therefore be compared on device-level metrics—capacity, life cycle, sensitivity, limit of detection, drift, and energy used per functional unit—together with precursor consistency and manufacturing yield. High-value, lower-volume products may justify more intensive purification when the added functionality and market value exceed the associated environmental burden.

### 8.5. Coatings, Composites, and Advanced Functional Materials

Colloidal, fumed, and porous silica are widely used to control rheology, reinforce polymer and cementitious matrices, improve abrasion resistance, create barrier layers, and generate hydrophilic or superhydrophobic surfaces. Particle size, aggregation state, surface silanol density, and compatibility with the surrounding matrix determine whether silica acts as a reinforcing phase, thixotropic additive, optical modifier, or surface-texturing component. These uses demonstrate that high-value function can arise from interfacial control even when internal porosity is not the dominant design variable.

Silica nanoparticle coatings and hybrid films have shown protective, self-cleaning, and superhydrophobic behavior [152,153,154]. Sustainable silica can be particularly attractive for these applications because some formulations tolerate broader impurity ranges than pharmaceutical or electronic systems, thereby reducing purification intensity. Nevertheless, color, refractive index, dispersion stability, surface roughness, mechanical adhesion, weathering, and compatibility with binders must remain consistent across batches.

Evidence is emerging for construction materials made with nanosilica extracted from rice husk ash; recent work on green ultra-high-performance concrete reports the use of RHA-derived nanosilica as a reactive mineral component [155]. This supports sustainable silica use in cementitious matrices. By contrast, the evidence base is still thinner for waste-derived colloidal silica specifically formulated as a post-applied concrete coating or surface modifier. This distinction should be retained so that established bulk/additive applications are not conflated with less-developed coating routes.

Coatings and composite additives represent an important bridge between bulk-volume and specialty markets. Construction and polymer applications can absorb substantial material quantities, whereas optical, protective, or multifunctional coatings command higher value but require narrower specifications. Investment strategies should therefore match feedstock quality and process sophistication to the intended market tier, using lower-complexity routes for qualified bulk applications and more controlled synthesis and functionalization for specialty products.

### 8.6. Biomedical and Pharmaceutical Systems

Biomedical and pharmaceutical applications provide a stringent but not exclusive test of silica design. Mesoporous silica can load poorly soluble drugs, biomacromolecules, or imaging agents through confinement and surface interactions, while pore size, connectivity, surface charge, and gating chemistry regulate release [2,19,20,21,156]. Drug loading should be evaluated together with solid state, pore blocking, release completeness, storage stability, and the dissolution of both cargo and silica. Apparent sustained release must be distinguished from inadequate sink conditions, aggregation, or sedimentation.

Biological performance depends on crystallinity, particle and aggregate size, aspect ratio, porosity, silanol density, charge, coatings, dose, route, and degradation rate [22,23,24,37,55,56,62,63,64,157,158,159,160]. Larger mesoporous particles often accumulate in liver and spleen after intravenous administration, whereas ultrasmall, carefully coated core–shell silica particles have demonstrated renal clearance and clinical imaging feasibility [159,161]. These examples confirm that architecture and surface chemistry can be engineered for biological fate, but they should not be generalized across particle classes or dosing regimens.

Biomedical use of sustainable silica is justified only when feedstock traceability, elemental impurities, residual templates and solvents, microbial quality, endotoxin, particle distributions, surface ligand density, degradation products, and batch comparability satisfy the requirements of the intended dosage form [162]. Moderate-purity materials may be suitable for selected non-systemic or excipient applications, whereas parenteral and diagnostic products require substantially tighter control. The pharmaceutical example therefore reinforces a principle applicable across this review: the acceptable synthesis route and purification intensity must be defined by the exposure scenario and critical quality attributes, not by precursor origin alone.

One boundary should be stated explicitly rather than left implicit. Silica hydrogels for injection, wound dressing and tissue scaffolding are an active field, but almost all reported systems use purified alkoxysilanes or commercial colloidal silica rather than waste-derived precursors, and the reason is regulatory rather than chemical. Parenteral and implantable products fall under the elemental impurity limits of ICH Q3D, which places arsenic, cadmium, mercury and lead in the class that must be assessed in every product, with permitted daily exposures in the microgram range [163]. Rice husk ash, bagasse ash, coal fly ash and geothermal scale each carry one or more of these elements at concentrations that require demonstrated removal rather than assumed absence, and geothermal silica carries arsenic specifically. Food-contact use faces a separate constraint: the European re-evaluation of silicon dioxide (E 551) was unable to confirm the previous acceptable daily intake and identified particle size distribution and the nanoscale fraction as data gaps that apply to any new source of synthetic amorphous silica [164,165]. The defensible position is therefore that waste-derived silica suits environmental, agricultural, construction, catalytic and coating applications now; that oral pharmaceutical excipient use is achievable with documented purification and batch release data; and that injectable and implantable gels remain out of reach until feedstock-specific impurity clearance is demonstrated batch by batch. Presenting the biomedical literature on alkoxide-derived silica hydrogels as though it transfers directly to waste-derived precursors would misrepresent the state of the field. 

**Table 6:** Selected applications of sustainable silica nanostructures and the structure–function relationships that govern their use. The table is not restricted to porous nanosilica; it includes dense colloidal, fumed/aggregated, porous, hybrid, and surface-functionalized platforms where these are relevant to the application. Examples are representative rather than direct rankings because feedstock composition, purification, architecture, test conditions, dose, and analytical methods differ among studies.

Application Field	Representative Sustainable Silica Platform	Structure–Function Basis and Selected Use	Key Qualification Need and References
Environmental remediation and separation	Fly ash-, rice husk-, or sodium silicate-derived porous and functionalized silica	Accessible pores and ligand chemistry support metal-ion, dye, pharmaceutical, and organic-contaminant capture	Realistic matrices, capacity, selectivity, regeneration, and leaching [15,109,110,115,131,132,138,139,166,167]
Catalysis and chemical processing	Rice husk- or fly ash-derived porous silica supporting metals or molecular catalysts	High-area interfaces disperse active phases and regulate transport, acidity, and recyclability	Matched active loading, turnover, selectivity, deactivation, and metal leaching [58,59,60,61,140,141]
Agriculture and controlled delivery	Rice husk ash SiNPs and porous silica carriers	Reactive silicon and tunable pores support stress mitigation and controlled nutrient or pesticide release	Field validation, dose, soil fate, runoff, and non-target effects [142,143,144,145,146,147,148,149]
Energy and electrochemical systems	Porous silica templates, coatings, catalyst supports, and silica-derived electrode materials	Pore architecture and interface control influence ion transport, phase dispersion, and cycling stability	Capacity or activity normalized to mass and energy, life cycle, impurity effects, and process yield [3]
Sensing and analytical platforms	Ligand-functionalized mesoporous silica and hybrid reporter systems	Molecular recognition, preconcentration, controlled diffusion, and reporter immobilization	Selectivity, interference, response stability, reproducibility, and regeneration [131,132,133,134,135,152]
Coatings, composites, and advanced materials	Colloidal, fumed, porous, or hybrid sustainable silica	Particle dispersion and interfacial chemistry control rheology, reinforcement, barrier properties, and surface texture	Matrix compatibility, weathering, adhesion, optical quality, and scalable dispersion [152,153,154]
Biomedical and pharmaceutical systems	Rice husk- or sodium silicate-derived mesoporous silica and ultrasmall core–shell systems	Pore confinement, surface chemistry, and degradability regulate loading, release, imaging, and biological fate	Purity, sterility, endotoxin, pharmacokinetics, degradation, and regulatory comparability [19,20,21,22,23,24,37,55,56,62,63,64,156,157,158,159,160,161,162]

Note: Quantitative outcomes such as removal efficiency, catalytic yield, crop response, loading, electrochemical performance, or biological effect must be interpreted using the conditions, uncertainty, number of replicates, and reference materials reported in the original study.

### 8.7. Cross-Application Qualification, Scale-Up, and Investment Relevance

The application examples collectively show that there is no universally optimal sustainable silica. Environmental sorbents prioritize accessible binding sites, regeneration, and low cost; catalysts require controlled interfaces and resistance to leaching; agricultural materials require field efficacy and environmental compatibility; energy and sensing systems demand precise electrochemical or analytical behavior; coatings require dispersion and durability; and biomedical products require the most stringent impurity, sterility, and biological-fate controls. A tiered specification strategy is therefore more rational than applying a single purity or particle size target to every market.

A tiered application strategy also clarifies investment potential. High-volume outlets such as treatment media, agricultural inputs, construction additives, and selected coatings can support regional plants located near concentrated waste streams. Higher-value markets—including catalysts, analytical materials, sensors, advanced coatings, and biomedical products—may justify additional purification, architectural control, and functionalization. A diversified product portfolio can reduce dependence on a single market, but each product must have a defensible specification, validated performance, and a credible route for feedstock supply, quality control, and end-of-life management.

Market segmentation also changes the appropriate basis for economic and environmental comparison. A regional bulk product and a tightly specified specialty material cannot be assessed using the same value proposition or functional unit; Section 9 therefore evaluates cost and life cycle claims against the service and specification actually delivered.

## 9. Life Cycle Assessment and Sustainability Metrics

Claims of lower environmental burden for waste-derived silica require the same scrutiny as claims of material performance. Life cycle assessment (LCA) and techno-economic analysis (TEA) should therefore be introduced as design tools rather than as end-stage validations. The outcome depends on feedstock allocation, pretreatment and purification intensity, electricity and heat supply, transport, reagent recovery, washing and drying, product yield, off-specification material, waste treatment, and the functional unit used to define equivalence [38,39,40,123,168,169].

The principal methodological difficulty is comparability. A kilogram of low-purity precipitated silica is not functionally equivalent to a kilogram of monodisperse, mesoporous, hydrophobized, or pharmaceutical-grade material. A defensible assessment should therefore report both mass-based results and, where possible, a performance-normalized functional unit such as adsorption capacity retained over cycles, catalyst productivity, thermal resistance, coating service life, or drug-loading/release performance. System boundary, waste allocation method, electricity mix, transport distance, acid/alkali recovery, wash-water treatment, drying route, silicon recovery, and product rejection rate should be varied in sensitivity analysis rather than held as hidden assumptions [168,169].

### 9.1. Life Cycle Assessment Frameworks and Comparative Environmental Impacts

Published LCAs support the possibility of substantial reductions in selected impact categories, but they do not justify a single universal carbon footprint value for ‘waste-derived silica’. Industrial precipitated nanosilica analysis shows that electricity, steam, alkali, and acid use can dominate cradle-to-gate impacts and that cleaner energy scenarios markedly change the result [168]. Waste-derived case studies can report lower burdens because precursor manufacture or disposal is avoided, but the magnitude depends on whether upstream burdens are allocated to the residue and on the additional purification required to reach the target specification [39,169].

A second limitation is the maturity of the inventory. Reviews of nanomaterial LCA have identified incomplete life cycle inventories, inconsistent treatment of end-of-life, limited toxicity characterization, and insufficient uncertainty and sensitivity analysis as persistent weaknesses [169]. These limitations are particularly important for emerging silica routes because laboratory energy use, solvent recycling, wash-water treatment, and scale-up yields are often estimated rather than measured. Consequently, early-stage LCA should be presented as prospective decision support with explicit uncertainty, not as a definitive environmental ranking.

The often-cited 80–98% greenhouse gas reduction range should therefore be treated as a favorable scenario envelope rather than a general property of secondary silica. Differences in this magnitude can arise when a burden-free waste allocation, low-carbon electricity, avoided-disposal credits, high silicon recovery, short transport, and limited purification are combined. More conservative allocation, fossil-intensive heat, repeated acid washing, solvent exchange, low product yield, or stringent high-purity specifications can substantially narrow the advantage. The revised comparison therefore avoids presenting 80–98% as a transferable estimate and instead emphasizes the conditions under which a reduction is robust.

Allocation is especially consequential. Treating a residue as burden free may be appropriate in one decision context, whereas mass, economic, or system expansion approaches assign part of the upstream process to that residue. None are universally correct; the choice must follow the goal and scope of the study and should be tested explicitly. The same applies to avoided-disposal credits, which depend on the realistic counterfactual fate of the residue. A strong LCA should report the conclusion under at least one conservative alternative allocation rather than only under the most favorable case [123,169].

Transport and site context add another layer of uncertainty. Agricultural residues may be geographically dispersed, while geothermal and industrial residues are concentrated but site specific; coal-derived feedstocks also face changing future availability. The relevant question is therefore not whether transport matters but at what distance or feedstock density the advantage over the comparator disappears under the study’s own assumptions. Reporting such break-even conditions is more informative than transferring a single carbon value from one region to another [39,40,169].

The methodological requirements outlined above explain why the studies summarized in Table 7 cannot be converted into a single ranked carbon footprint series. The table instead shows how system boundary, inventory maturity, allocation, regional context, and functional equivalence shape the conclusion; any route-specific reduction remains conditional on those assumptions.

Environmental advantage and economic advantage should be evaluated together but not conflated. A waste-derived route may begin with negligible feedstock cost yet become expensive through purification, solvent exchange, low silicon recovery, corrosion control, or wastewater treatment; conversely, a route with higher reagent cost may remain competitive if it improves yield, shortens processing, or reaches a higher-value specification. Table 8 therefore complements the LCA evidence in Table 7 by showing indicative production cost ranges and the process conditions that can shift them, rather than providing a separate ranking of precursor classes.

### 9.2. Circular Economy Integration and Challenges

Circularity claims should distinguish simple waste diversion from genuine system-level value retention. The relevant questions are whether a route reduces virgin inputs, preserves or upgrades material value, creates usable co-products, and avoids transferring contaminants or treatment burdens into a new product or effluent stream.

Several closed-loop concepts have been proposed. Rice husk-derived silica can be returned to agricultural value chains through silicon-containing soil amendments or deliberately synthesized silicon–potassium fertilizers [150,156]. Rice husk-derived silica gel has also been used in fly ash inertization under microwave treatment [41]. Geothermal waste-derived nanosilica has been investigated for enhanced oil recovery [107], while fluorosilicic acid can be converted to high-surface-area mesoporous silica [105]. These examples are consistent with broader circular economy principles that emphasize resource recovery and system-level value retention [42].

Circularity should also be assessed beyond the first conversion step. Regeneration or end-of-life fate may determine whether a silica adsorbent, catalyst support, agricultural carrier, or pharmaceutical material closes a resource loop or simply shifts burden. Potential pathways include adsorbent regeneration, recovery of captured metals, reuse of catalyst supports, incorporation of spent silica into cementitious materials, and safe dissolution or excretion of biomedical silica. The preferred route must account for contaminants accumulated during use and avoid downcycling hazardous residues into uncontrolled applications.

## 10. Critical Assessment, Standardization, and Future Outlook

This section consolidates the implications of the preceding chemistry, process, application, and systems analyses. The remaining challenge is no longer proof that secondary silicon can form functional silica, but establishing the control rules, reporting standards, and validation sequence needed to determine when a route is genuinely preferable and ready to scale.

### 10.1. Fundamentals of Performance Comparison: Are Sustainable Precursors and Methods Better?

The comparison that matters is not between waste-derived and alkoxide-derived silica in general, but between specific combinations of source and process measured against a defined product specification. Rice husk ash and a refined bio-based precursor illustrate the two extremes. Rice husk ash is abundant, effectively free, and available wherever rice is milled, but its silica content, alkali load, and crystallinity depend on cultivar, soil, and combustion history, so every batch requires characterization and the extraction conditions must be adjusted rather than fixed. A refined precursor prepared from the same biomass, such as a purified silicate solution or an alkoxide synthesized from biogenic silicon, restores reproducibility and removes the trace metal problem, but it reintroduces the processing energy and reagent consumption that made the alkoxide route expensive in the first place. Neither is universally preferable. The abundant source is the rational choice where impurity tolerance is high and volumes are large, as in construction additives, soil amendments, and bulk adsorbents. The refined precursor is the rational choice where the specification is tight and volumes are small, as in chromatographic media and pharmaceutical excipients. The intermediate cases, which are the majority, require an explicit decision about how much purification to buy.

That decision is difficult because the purity-versus-energy trade-off is nonlinear. Removing the first fraction of impurity from an ash-derived silicate is inexpensive, since a single acid wash and a water rinse eliminate most of the alkali and much of the calcium. Removing the last fraction is expensive, because the remaining trace metals are incorporated into the silica network rather than adsorbed on it, and their removal requires redissolution and reprecipitation with the reagent and effluent burden that this implies. Cost and environmental burden therefore rise steeply as the specification tightens, while the performance gain frequently saturates well before analytical purity is reached. A design rule follows directly: identify the impurity level beyond which the intended function no longer improves, and purify to that level rather than to the highest level achievable. Reporting purity as a headline figure without the corresponding reagent, water, and energy intensity conceals precisely the variable on which the sustainability claim depends.

Three fundamental questions remain open and should organize the next phase of work. The first is the quantitative relationship between a measured feedstock composition and the resulting gelation behavior, which cannot currently be predicted for any waste source and which requires controlled single-variable studies rather than further feedstock demonstrations. The second is the structural role of retained impurities in the network. Aluminum, calcium, and residual carbon are known to modify connectivity, dissolution rate, and pore development, but the relationship between their concentration and the Q^n^ distribution has not been established, so the possibility of using them deliberately as network modifiers remains unexplored. The third is scale-up kinetics, where the interaction between micromixing, local supersaturation, and gel time governs whether a laboratory result survives a hundredfold increase in volume, and where almost no data exist for waste-derived silicates. Progress on these three questions would convert sustainable silica synthesis from a collection of demonstrations into a design discipline.

### 10.2. Terminology Harmonization and Standardization

One of the fundamental challenges in the sustainable silica field is the lack of consistent terminology, reporting practices, and evaluation criteria. Although silica nanoparticles encompass a broad range of materials with different architectures and properties, terms such as “mesoporous silica nanoparticles,” “nanosilica,” and “sol–gel silica” are frequently used without clearly defining structural characteristics, synthesis conditions, or quality parameters. Similarly, the relationship between classical approaches, such as Stöber synthesis, general sol–gel processing, and waste-derived silicate precipitation routes, is not always explicitly distinguished, making direct comparison between studies difficult.

Inconsistent reporting extends beyond terminology to experimental methodology. Differences in precursor composition, extraction procedures, purification strategies, reaction parameters, aging conditions, drying methods, and characterization protocols often result in materials with substantially different properties despite being described using similar terminology. Such variations limit reproducibility and prevent meaningful evaluation of whether a reported improvement originates from the synthesis route itself or from differences in material characterization.

The establishment of standardized reporting frameworks is therefore essential. At minimum, studies should define feedstock origin and sampling, proximate and elemental composition, silica phase and content, pretreatment history, reagent grades, solid-to-liquid ratios, silicon recovery, pH trajectory, temperature, mixing and addition conditions, aging and drying, washing intensity, final yield, particle size distribution in dry and dispersed states, surface area and pore size methodology, Q^n^ connectivity where relevant, elemental impurities, residual organics or surfactants, and uncertainty or batch-to-batch variability. The comparisons should distinguish measured values from literature assumptions and report units, sample number, standard deviation, and detection limits where applicable. ISO/TR 13014 provides a useful general basis for physicochemical characterization in toxicological assessment, but silica-specific, application-linked acceptance criteria remain necessary [170].

Regulatory harmonization is equally important for products involving human exposure or environmental release. Existing precedents for specified silica forms and uses—including Generally Recognized as Safe (GRAS) status in defined U.S. food use contexts—do not automatically transfer to every nanoscale or waste-derived material. Particle characteristics, surface chemistry, exposure route, impurity profile, and intended use determine the evidence required. Regulatory acceptance should therefore be framed as application- and product-specific comparability rather than as a blanket property of SiO_2_ [171].

### 10.3. Scalability, Regulation, and Data Gaps

Scale-up shifts the dominant risks from chemical feasibility to feedstock qualification, micromixing and residence time control, separation efficiency, fouling and corrosion, and waste management. Feedstock variability matters because it changes these process responses, not simply because the source is a waste. Scale-up studies should therefore demonstrate that defined product specifications are maintained across representative feedstock lots and operating campaigns [35].

The most credible scale-up pathway combines feedstock qualification with modular processing. Incoming feedstocks should be classified by silica phase, impurity fingerprint, moisture, and reactivity; blending or adaptive recipes can then reduce seasonal variability. Continuous alkaline extraction and controlled neutralization can improve residence time control, while membrane separation, counter-current washing, and solvent or reagent recovery may lower water and salt burdens. Digital mass balance and statistical process control should be incorporated early, not added after scale-up. Pilot studies must report productivity, fouling, cleaning, equipment corrosion, off-specification material, and wastewater composition because these factors often dominate industrial feasibility.

The regulatory position is more defined than the literature sometimes implies. Synthetic amorphous silica is managed within established chemical and product frameworks, including the European Union Registration, Evaluation, Authorisation and Restriction of Chemicals (REACH) system for relevant industrial uses. For food use, silicon dioxide (E 551) is authorized under defined conditions, while European re-evaluation has emphasized characterization of the nanoscale fraction [164,165]. For pharmaceutical products, ICH Q3D governs elemental impurities [163], and parenteral or device applications add sterility/endotoxin and product-specific requirements. None of these frameworks excludes a secondary silica source in principle; they shift the burden to batch-level evidence of composition, particle attributes, purity, and comparability. This is why regulatory and analytical requirements are introduced in the review before biomedical application claims rather than being treated solely as a final commercialization step.

Safety assessment for applications involving human or environmental exposure should begin before formulation lock and remain tied to the product’s critical quality attributes and exposure route. Section 10.4 develops this principle into a staged safe-and-sustainable-by-design research priority.

**Figure 6:**
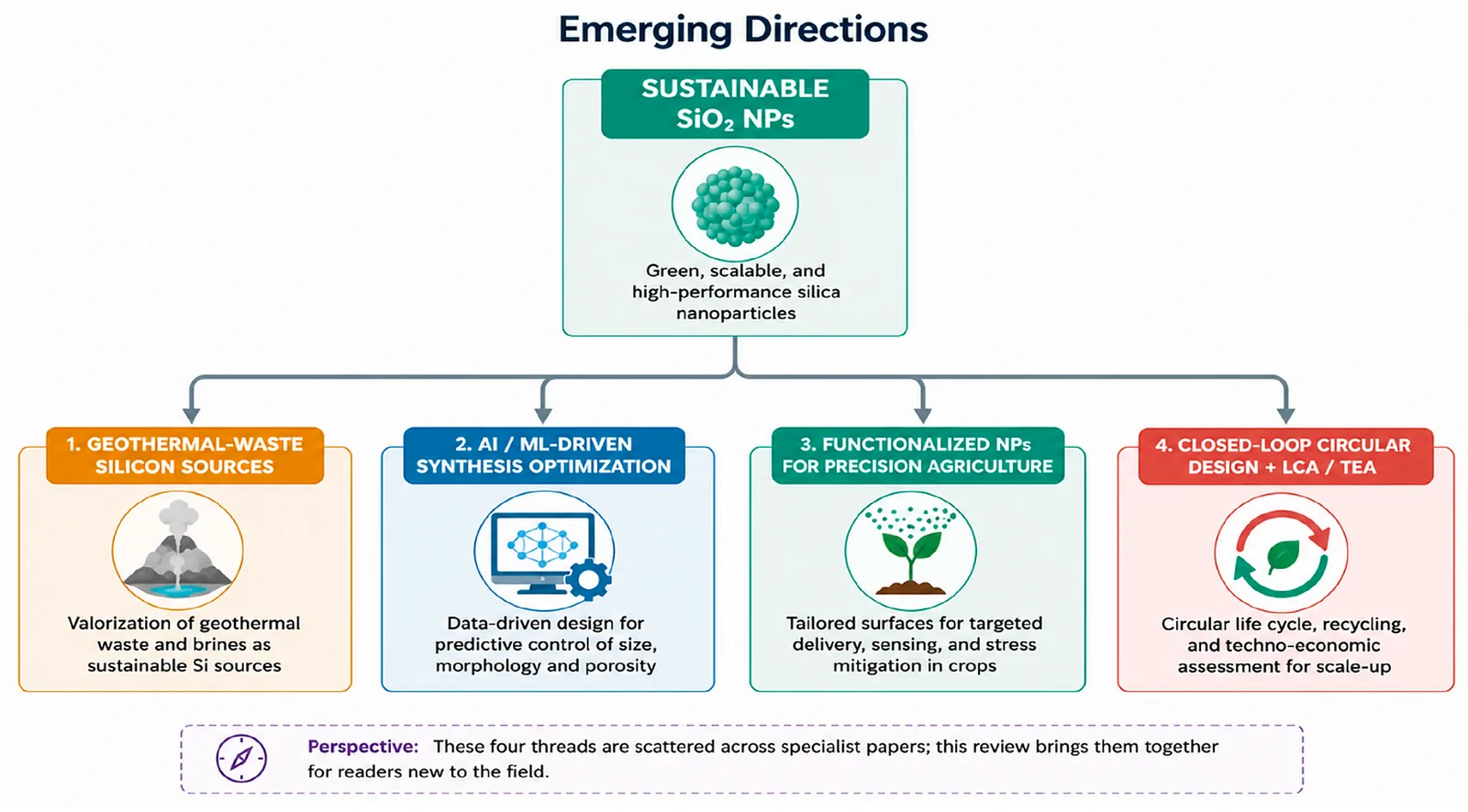
Emerging research directions for sustainable silica production. The roadmap integrates feedstock qualification, low-temperature and continuous synthesis, process analytical technology, data-driven optimization, impurity-aware structure control, green functionalization, application-specific safety assessment, circular resource integration, and scale-up. Progress requires quantitative links between process parameters, critical material attributes, functional performance, and life cycle outcomes.

The priorities emerging from these regulatory, analytical, and scale-up constraints are consolidated in Figure 6. The roadmap emphasizes feedstock qualification, lower-burden processing, process analytical control, data-driven optimization, impurity-aware design, application-specific safety, circular resource integration, and scale-up as mutually dependent research directions.

**Figure 7:**
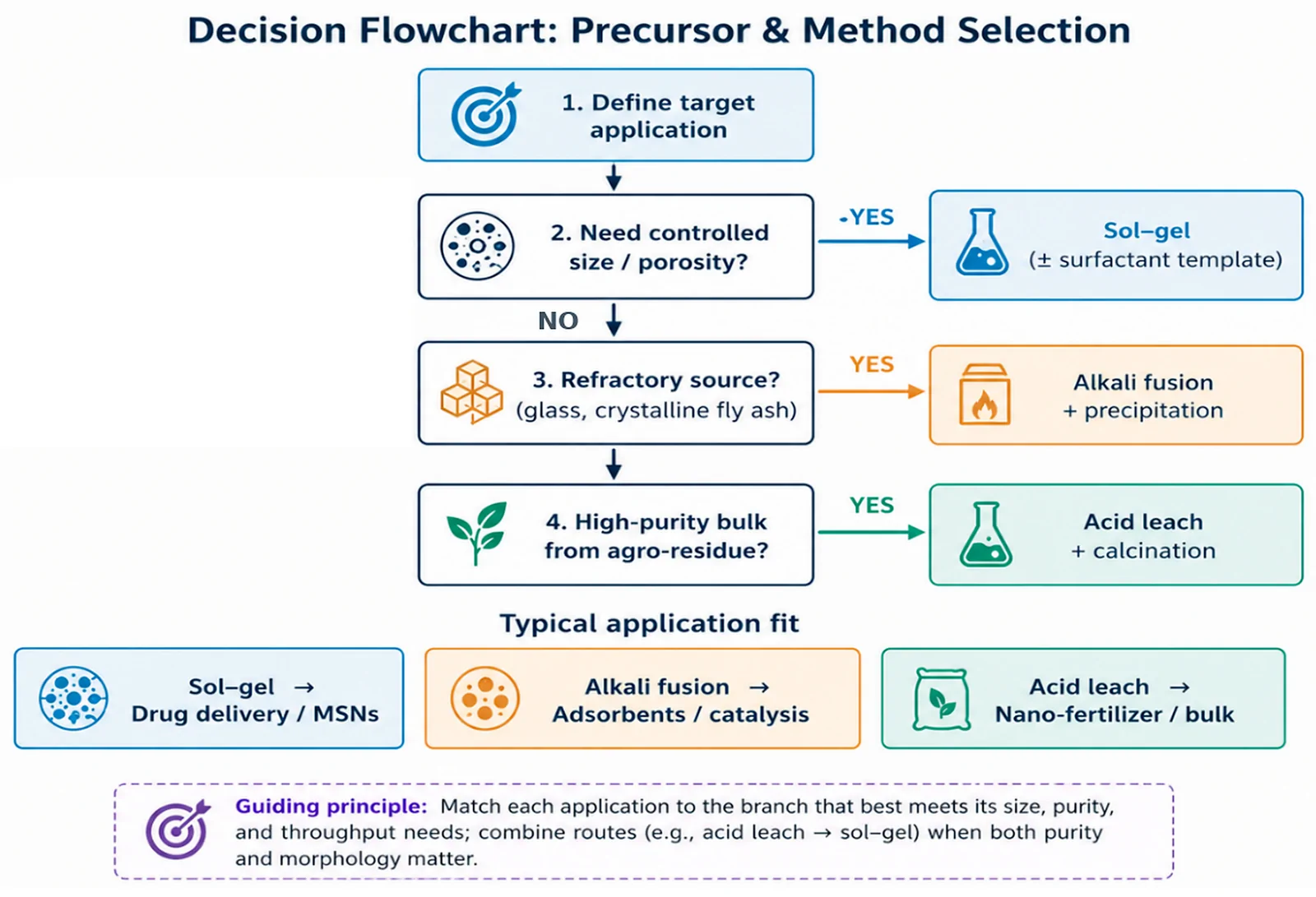
The decision framework for precursor and processing route selection. Feedstock silica content, phase, impurity profile, geographic availability, and variability are matched to the required purity, particle size, porosity, surface chemistry, production scale, and exposure scenario. If tight size/porosity control is not required, the decision proceeds directly to feedstock refractoriness and then to application-specific pretreatment. The redundant left-side ‘No’ box has been removed to make the sequence explicit.

Translating these priorities into practice requires a structured choice among feedstock quality, purification intensity, synthesis route, and the specification of the intended product. Figure 7 therefore provides a stepwise decision framework for selecting a precursor and processing route according to feedstock composition, required particle attributes, production scale, and exposure scenario.

### 10.4. Research Priorities and Further Directions

Future work should move from additional proof-of-concept conversions to hypothesis-led programs that close a defined causal link in the feedstock-to-function chain. The most urgent priorities are quantitative impurity-response maps, representative feedstock sampling, scale-up kinetics, closed-loop process intensification, performance-normalized sustainability assessment, and application-specific safety. Each program should include a predefined comparator, replicated batches, uncertainty reporting, and prospective validation rather than relying on a single optimized endpoint.

Quantitative impurity response maps. A rigorous design should begin with a compositionally defined alkali silicate matrix and a screening experiment in which Al, Ca, Mg, Fe, Na/K excess, residual carbon, and application-relevant trace elements are varied over concentrations spanning the low, median, and high ranges measured in authentic feedstocks. A fractional–factorial or definitive-screening design can identify dominant main effects and interactions; the influential variables should then enter a response surface or mixture design with replicated center points, randomized run order, and at least three independently prepared baseline silicate batches. Predefined responses should include induction and gel times, storage/loss moduli, DLS size distribution and polydispersity, SAXS descriptors where available, Q^n^ populations by ^29^Si NMR, silicon recovery, shrinkage, BET area, pore size distribution, and dissolution. Models should report effect sizes, interaction terms, confidence or prediction intervals, lack-of-fit testing, and sensitivity to analytical uncertainty. Finally, the response surface should be challenged prospectively with at least three chemically distinct authentic feedstock lots that were not used to fit the model. The output is a validated impurity–process–structure map, not another table of final BET values [37,43,44,46].

Feedstock qualification, sampling, and digital traceability. A representative sampling protocol is needed for each major resource class because seasonal, geographic, combustion, and storage variability cannot be corrected by a single analysis of one batch. Future studies should quantify within-lot and between-lot covariance in silica phase, loss on ignition, moisture, alkali and alkaline earth content, trace elements, and dissolution reactivity. Rapid reactivity tests and multivariate compositional fingerprints should be linked to blending rules and adaptive extraction recipes. A digital feedstock passport recording origin, processing history, composition, reactivity, and uncertainty would provide the traceability required for statistical process control and regulated products [171,172].

Scale-up kinetics and reactor engineering. Scale-up studies should report mixing time, acid or base addition profile, local and bulk pH, temperature gradients, residence time distribution, solids loading, shear history, productivity, and off-specification fraction. Computational fluid dynamics and dimensionless analysis should be coupled with experimentally measured nucleation and gelation kinetics to identify when micromixing becomes slower than condensation. Continuous stirred-tank cascades, plug-flow neutralization, oscillatory baffled reactors, and modular membrane-assisted washing should be compared against batch processing at equivalent product specifications. Process analytical technology should monitor conductivity, turbidity, particle size, rheological state, and silicon concentration in real time, while pilot campaigns should document fouling, corrosion, cleaning, start-up, shutdown, and material losses.

Closed-loop process intensification. Research on low-temperature extraction, microwave heating, mechanochemical activation, ambient-pressure drying, and green solvent systems should include complete mass and energy balances. Reduced reaction time is not sufficient if the route increases alkali excess, solvent exchange, washing volume, silylating agent consumption, or effluent treatment. Priority should be given to acid and alkali regeneration, counter-current washing, membrane concentration, electrodialysis, solvent recovery, heat integration, and conversion of sodium, calcium, fluoride, or alumina streams into useful co-products. The decisive metric is the burden per unit of silica meeting a defined specification, including rejected batches and recovered reagents, rather than the energy consumed during the nominal synthesis step alone.

Mechanism-informed data infrastructure and machine learning. Data-driven optimization will remain unreliable while studies report different feedstock descriptors, omit negative results, and collapse pore structure or particle size into single mean values. A Findable, Accessible, Interoperable, and Reusable (FAIR), machine-readable dataset should capture feedstock composition, silicate modulus and concentration, pH trajectory, mixing and addition conditions, gelation metrics, aging, washing, drying, surface modification, uncertainty, and functional performance. Physics-informed and small-data methods should then be used to interpolate within chemically defined domains, identify influential variables, design the next experiment, and quantify prediction uncertainty [173,174,175,176]. Models should be tested prospectively on unseen feedstock lots and external laboratories; retrospective goodness of fit alone is not evidence of process predictability.

Performance-normalized life cycle and techno-economic assessment. Building on Section 9, future assessments should use functional units that reflect the service delivered, such as adsorption capacity retained over regeneration cycles, catalyst productivity over lifetime, thermal resistance, coating durability, or drug-loading and release performance. Regional and prospective scenarios should test electricity supply, transport distance, feedstock allocation, avoided-disposal credits, water scarcity, reagent recovery, product yield, and scale, with uncertainty and sensitivity analysis used to identify when an apparent advantage is robust [38,39,40,123,168,169]. TEA and LCA should be updated at laboratory, pilot, and pre-commercial stages so that purification and architecture decisions are evaluated before they become locked into equipment design.

Safe-and-sustainable-by-design qualification. Safety assessment should be matched to the exposure route and initiated while the material and process remain adjustable. Studies should distinguish intact particles from dissolved silicon and detached labels, quantify elemental and organic leachables, and examine how surface silanols, aggregation, porosity, and framework condensation affect biological and environmental fate. Tiered programs should combine dissolution and leaching, colloidal stability, membrane and hemolysis testing where relevant, immune and oxidative stress endpoints, environmental transformation, and longer-term distribution and clearance [22,23,24,157,158,162,163,164,165]. Critical quality attributes, reference standards, and comparability criteria should be agreed early with regulatory and end-user stakeholders rather than reconstructed after the synthesis route has been fixed.

Benchmark materials, interlaboratory studies, and staged pilot validation. The field would benefit from a small set of reference feedstocks and products spanning low, intermediate, and high impurity tolerance. Examples could include a bulk adsorbent, a catalyst support or coating additive, and a high-purity porous carrier. Participating laboratories should process common feedstocks using harmonized protocols, report the minimum dataset defined in this review, and compare both material attributes and functional performance. Interlaboratory variance, rather than the best single result, should determine whether a method is mature enough for pilot manufacture. Pilot validation should proceed through explicit gates for silicon recovery, productivity, batch consistency, reagent and water intensity, waste treatment, safety, and end-user performance [170].

A future study should therefore be considered decisive only when it closes at least one causal link in the feedstock-to-function chain and reports the associated uncertainty. The strongest contributions will predict a process outcome from a measured feedstock property, demonstrate control at a larger or continuous scale, or show that a lower-burden route delivers equivalent function under a transparent system boundary. This standard would reduce repetitive feasibility studies and redirect effort towards the knowledge required for reliable design and adoption.

## 11. Conclusions

Sustainable silica nanoscales synthesis is best understood as established silica chemistry operating from more variable starting states. Agricultural residues, industrial by-products, geothermal resources, and fluorosilicate streams do not alter the fundamental Si–O–Si network-building chemistry; they alter silicon speciation, impurity environment, dissolution requirements, and the kinetics through which the network forms. The central design task is therefore to match feedstock chemistry and process history to the critical material attributes required by a defined application.

Emerging processing strategies make matching more practical by addressing specific bottlenecks. Ambient-pressure drying shifts the aerogel problem from pressure equipment to solvent exchange and surface modification; microwave and mechanochemical activation alter heating or dissolution kinetics; biogenic templating changes interfacial assembly; and continuous processing addresses mixing and residence time control. Their value should be judged by the burden removed, the burden introduced, and whether structural control is retained.

The evidence reviewed here shows that secondary resources can yield silica with competitive purity, architecture, and function across environmental, catalytic, agricultural, coating, energy, sensing, and selected biomedical applications. Waste-derived aerogels reaching approximately 350–500 m^2^ g^−1^ illustrate that there is no demonstrated intrinsic performance ceiling imposed by feedstock origin. The persistent limitation is reproducibility: impurities, colloidal state, gelation, aging, washing, and drying must be controlled well enough to reproduce the required pore structure and surface state.

Translation therefore requires a common evidence chain: feedstock traceability and representative sampling; intermediate silicate and colloidal characterization; silicon mass balance; gelation and aging metrics; fit-for-purpose impurity limits; application-specific performance and safety criteria; performance-normalized LCA and TEA; process analytical control; and staged pilot validation. Sustainable silica should be judged by function and control within a transparent system boundary, not by precursor substitution alone. This criterion directs the field away from repeated feasibility demonstrations and toward predictable, scalable, and commercially credible manufacturing.

Future progress depends on making that shift operational. Once the relationship between feedstock composition and network formation is quantified, the impurities that are presently treated as contamination become adjustable parameters, and a waste stream becomes a designable precursor rather than a cheap substitute. Aluminum could then be dosed to control dissolution rate in a biodegradable carrier, residual carbon could be used as a sacrificial template rather than washed away, and fluoride recovered from fertilizer processing could direct mesostructure at ambient temperature. Achieving this requires no new chemistry. It requires that gelation, aging, and drying be measured with the same rigor that has long been applied to alkoxide systems, and that feedstock composition be reported as a process variable rather than as a purity claim. Such a framework would allow sustainable silica synthesis to progress from demonstrating that waste can be converted to specifying, in advance, what a given waste can be converted into.

## Figures and Tables

**Table 1:** Reported textural properties of silica aerogels prepared from secondary silicate precursors by ambient-pressure drying. Values are reported as in the original studies and are not directly comparable because aging, solvent exchange, and surface modification differ. Trimethylchlorosilane (TMCS) and hexamethyldisilazane (HMDS) are common silylating agents. The rice-hull-ash route that adds TEOS before gelation is explicitly classified as TEOS-supplemented rather than alkoxide-free.

Feedstock	Route and Drying Method	BET Surface Area (m^2^ g^−1^)	Mean Pore Diameter (nm)	Pore Volume (cm^3^ g^−1^)	Reference	Route Class
Sugarcane bagasse ash	Alkaline extraction to sodium silicate; solvent exchange; TMCS/HMDS silylation; ambient-pressure drying	441	3.8	0.75	[49]	Alkoxide-free
Rice hull ash	Alkaline extraction; H_2_SO_4_ neutralization; TEOS added to the sol; ethanol exchange; ambient-pressure drying	≈500	26.5	3.31	[51]	TEOS-supplemented
Coal fly ash	Alkaline extraction; silylation; ambient-pressure drying	362	10.4	0.95	[52]	Alkoxide-free
Rice husk ash	Alkaline extraction to sodium silicate; surface modification; ambient-pressure drying	Reported in the same range as alkoxide-derived aerogels	Not resolved as a distribution	Not reported consistently	[53]	Alkoxide-free
Wheat husk ash	Alkaline extraction; silylation to a hydrophobic network; ambient-pressure drying	Comparable to bagasse ash aerogels	Mesoporous	Not reported consistently	[50]	Alkoxide-free

**Table 2:** Comparison of conventional and sustainable silica precursors. Values are indicative literature ranges and depend on feedstock composition, allocation, purification, energy mix, synthesis route, product specification, and scale. TEOS, tetraethyl orthosilicate; TMOS, tetramethyl orthosilicate; SiNP, silica nanoparticle; CO_2_-eq, carbon dioxide equivalent.

Parameter	TEOS/TMOS	Rice Husk Ash	Fly Ash	Geothermal Waste	Fluorosilicic Acid
Cost ($/kg)	50–200	2–5	0–2 (disposal savings)	0 (disposal savings)	0 (disposal savings)
SiO_2_ content (%)	100 (pure)	85–97	40–60	60–80	High (Si source)
Toxicity	High (irritant, flammable)	Low	Moderate (heavy metals)	Low	High (corrosive, fluoride)
Production energy	High (chemical synthesis)	Low (combustion by-product)	Minimal (waste-derived)	Minimal (waste-derived)	Minimal (waste-derived)
Achievable SiNP purity (%)	>99.9	95–99	90–99	95–99	95–99
Carbon footprint (kg CO_2_-eq/kg)	8–15	2–4	1–3	1–3	2–4
Global availability (t/yr)	Limited	>100 million	>750 million	Hundreds of thousands	5–6 million
Pretreatment complexity	None	Moderate (acid leaching)	High (impurity removal)	Moderate (purification)	Moderate (neutralization)
Waste valorization benefit	None	High	Very high	High	Very high

Note: Values are not directly comparable unless the same system boundary, functional unit, and product specification are used.

**Table 3:** Comparison of precursor classes by silicon speciation, rate-limiting step, nucleation behavior, dominant control variable, and characteristic product. The classification is chemical rather than by waste or virgin origin, because the form in which silicon enters the reaction predicts synthesis behavior more reliably than feedstock provenance does.

Precursor Class	Silicon Speciation on Entry	Rate-Limiting Step	Nucleation and Growth Behavior	Dominant Control Variable	Characteristic Product and Principal Constraint
Alkoxysilanes (TEOS, TMOS)	Single molecular species; alkoxy groups must be hydrolyzed before condensation	Hydrolysis	Homogeneous burst nucleation followed by diffusion-limited growth; nucleation and growth are separable in time	Water-to-silicon ratio, catalyst, temperature	Monodisperse dense spheres and ordered mesoporous particles; high precursor cost and upstream manufacturing energy
Alkali silicates (commercial or ash-derived water glass)	Molecular/oligomeric silicates with possible colloidal silica; acidification shifts equilibria toward Si(OH)_4_/silanols	Acidification-controlled Si(OH)_4_ formation, condensation, and colloidal destabilization	Nucleation, condensation, growth, and aggregation overlap; pre-existing colloids may seed or broaden the population	Acidification rate and mixing; silicate modulus; ionic strength; colloidal size distribution by DLS	Precipitated silica, colloids, xerogels, and aerogels; broader distributions and residual salts if poorly controlled
Solid amorphous silicates (rice husk and bagasse ash, fly ash, geothermal scale)	Silicon bound in an amorphous solid and released only on dissolution	Dissolution of the solid phase	Local supersaturation gradients; heterogeneous nucleation on residual carbon and mineral phases	Extraction temperature, alkali loading, particle size, combustion history	Same product range as alkali silicates at far lower feedstock cost; batch-to-batch variability and trace metals
Fluorosilicate solutions (H_2_SiF_6_)	Molecular fluorosilicate anion, already dissolved	Hydrolysis with concurrent fluoride release	Controlled nucleation with fluoride-mediated siloxane rearrangement, favoring order at low temperature	pH, temperature, rate of fluoride removal	High-purity and ordered mesoporous silica at low temperature; hydrogen fluoride handling and fluoride disposal

**Table 4:** Comparison of primary green synthesis routes for sustainable silica nanoparticles. Reported ranges vary with precursor phase, composition, reactor configuration, washing, drying, and post-treatment. Particle size refers to the reported product and may represent primary particles, aggregates, or agglomerates; direct comparison therefore requires consistent dispersion and measurement methods.

Method	Particle Size	Purity (% SiO_2_)	Energy Demand	Scalability	Key Advantages	Main Drawbacks
Acid leaching + calcination	1–100 µm (needs milling)	95–98	High	Excellent	Simple, low cost, basic equipment	Microparticles; acidic wastewater; high thermal energy
Alkoxide-based sol–gel (Stöber/templated; comparator)	20–500 nm (tunable)	>99	Low–moderate on-site; upstream precursor burden	Good	High control of particle size and porosity	High TEOS/solvent cost; upstream precursor manufacture; template/solvent removal
Alkali fusion + precipitation	50 nm micron	90–99	High (450–600 °C)	Good	Handles refractory sources; co-product recovery	High alkali use; energy-intensive; salt waste
Fluorosilicic acid hydrolysis	32–200 nm	95–99	Moderate; route-dependent	Moderate	Valorizes hazardous waste; high-purity silica	HF/fluoride management; corrosion control; product purification
Alkali silicate precipitation/sol–gel (commercial or waste-derived water glass)	20 nm micron; route dependent	90–99	Low–moderate	Good	Avoids alkoxide hydrolysis; compatible with secondary silicate liquors	Requires acidification/mixing control; salt washing; colloidal fraction must be characterized
Hydrothermal feedstock treatment/phase conversion	Route-dependent	Feedstock-dependent	Moderate–high	Moderate	Activates refractory solids and can support controlled phase transformation	Pressure/temperature duty; may form zeolitic or calcium silicate phases rather than silica; product identity required

Note: Energy demand categories are qualitative because studies use different boundaries and may exclude precursor production, wastewater treatment, or drying.

**Table 5:** Emerging strategies for sustainable silica synthesis, compared by mechanistic basis, highest reported demonstration scale, principal sustainability advantage, and principal barrier. Indicative technology readiness levels are the assessment of the present review, based on the highest demonstration scale reported in the literature rather than on a formal readiness audit; no independent TRL assignment exists for any of these routes, and the values should be read as relative rather than absolute.

Emerging Strategy	Mechanistic Basis	Highest Reported Scale (Indicative TRL)	Principal Sustainability Advantage	Principal Barrier
Continuous flow and microreactor processing	Segmented or tubular flow holds the local acid-to-silicate ratio constant and separates nucleation from growth	Laboratory to bench (3–4)	Reproducible gel time and narrow size distribution without additional reagent	Wall fouling by the growing gel; online gel point detection not yet demonstrated
Microwave-assisted activation	Direct dielectric coupling to water and hydroxylated silicate raises the condensation rate at lower bulk temperature	Laboratory (3–4)	Shorter cycles and lower thermal input; narrower size distribution	Penetration depth limits vessel size; field uniformity degrades on scale-up
Mechanochemical activation and solvent-free extraction	Milling introduces lattice defects and amorphizes crystalline phases, raising the dissolution rate of the solid	Laboratory to pilot for ash activation (4–5)	Reduces or removes the high-temperature fusion step; little or no solvent	Milling energy can offset the saving; contamination from grinding media
Room-temperature routes with organic acids and nonionic surfactants	Buffered acidification slows the approach to the isoelectric region and suppresses local supersaturation spikes	Laboratory (3–4)	Removes the hydrothermal step; milder reagents and lower corrosion duty	Longer processing times; mesostructural order inferior to hydrothermal templating
Biogenic and plant extract templating	Plant-derived polyelectrolytes and polyamines lower the interfacial energy of the forming mesophase	Laboratory (2–3)	Replaces synthetic surfactants; the template is itself a residue stream	Extract composition varies with source and season; template removal incomplete
Biomimetic and enzyme-mediated silicification	Silicatein and silaffin-type polycations catalyze siloxane condensation at neutral pH and ambient temperature	Laboratory proof of concept (2)	Ambient temperature and neutral pH; morphological control without surfactant	Enzyme cost and stability; not yet demonstrated on a waste-derived silicate
Deep eutectic and bio-based solvents	Hydrogen-bonded eutectic mixtures replace volatile organic solvents as reaction or exchange media	Demonstrated for functionalization rather than synthesis (2)	Low volatility and toxicity; recyclable; compatible with solvent exchange before drying	High viscosity limits mass transfer; product recovery; unproven as a synthesis medium for waste silicate
Machine learning-guided process optimization	Data-driven models map feedstock composition and process variables onto measured product attributes	Laboratory (3)	Reduces the experimental burden imposed by feedstock variability	Requires standardized, comparable datasets that the field does not yet produce

**Table 7:** Representative LCA approaches relevant to sustainable nanosilica and the assumptions that limit direct comparison across studies.

Study/Scope	Functional Unit and Boundary	Primary Analytical Emphasis	Main Limitation for Cross-Study Comparison
Klöpffer et al. [123]	General nanotechnology LCA framework; methodological rather than a single silica process	Goal/scope, inventory, system boundary, and functional equivalence	Provides framework guidance but no feedstock-specific nanosilica inventory
Nizam et al. [169]	Review of 71 nanomaterial LCA studies; multiple materials and boundaries	Inventory transparency, end-of-life, toxicity, uncertainty, and sensitivity analysis	Cross-material review; not a process-specific carbon value for silica
Gu et al. [168]	Industrial precipitated nanosilica; cradle-to-gate raw-material acquisition/transport through production	Electricity, steam, alkali, acid, and cleaner-energy scenarios	Regional industrial case; conventional precipitated route and electricity mix limit transferability to waste-derived systems
Dominic et al. [39]	Millet husk-derived nanosilica case study	Waste-derived extraction, characterization, and environmental assessment	Feedstock- and process-specific result; allocation, purification sequence, scale, and product specification limit generalization

**Table 8:** Indicative production cost ranges for conventional and waste-derived silica routes. Values depend on feedstock allocation, plant scale, silicon recovery, purification, reagent recycling, energy source, transport, drying, waste treatment, and product quality; they should be treated as scenario ranges rather than universal costs.

Route	Feedstock Cost	Reported Production Cost	Note
TEOS-based (conventional)	High (commercial precursor)	$20–50/kg	Standardized precursor; high energy and reagent cost
Rice husk ash sol–gel	$2–5/kg	$2–8/kg	Low feedstock cost; purification-dependent
Fly ash alkali fusion	0 to negative (disposal savings)	Low, purification-sensitive	Multi-stage purification raises cost
Geothermal silica	0 (disposal savings)	Low, site-dependent	Limited geography; arsenic control
Fluorosilicic acid hydrolysis	0 (disposal savings)	Moderate	HF/fluoride management adds cost

Note: TEA, techno-economic analysis; all monetary values are nominal US dollars as reported and are not inflation-adjusted.

## Data Availability

No new data were created or analyzed in this study. Data sharing is not applicable to this article.

## Glossary

Abbreviations and key terms used throughout this review.

**Term/Abbreviation**	**Definition**
**SiNP/SiO_2_NP**	Silica (silicon dioxide) nanoparticle
**MSN**	Mesoporous silica nanoparticle (typically a xerogel)
**TEOS**	Tetraethyl orthosilicate, a conventional alkoxysilane precursor
**TMOS**	Tetramethyl orthosilicate, a conventional alkoxysilane precursor
**RHA**	Rice husk ash, a high-silica agricultural residue
**FA**	Fly ash, a coal combustion industrial by-product
**FSA**	Fluorosilicic acid (H_2_SiF_6_), a phosphate-industry by-product
**Water glass**	Aqueous sodium or potassium silicate solution
**Sol–gel**	Hydrolysis and condensation route from a silicate or alkoxide sol to a gel network
**Xerogel**	Dried gel produced by ambient pressure drying (moderate surface area, porosity)
**Aerogel**	Dried gel produced by supercritical drying (high surface area, high porosity)
**Alkali fusion**	High-temperature reaction of a silica source with NaOH or Na_2_CO_3_ to form soluble silicate
**Calcination**	Heating in air to remove organics and convert material to oxide
**TEA**	Techno-economic analysis
**LCA**	Life cycle assessment
**CAPEX/OPEX**	Capital expenditure/operating expenditure
**GHG**	Greenhouse gas
**BET**	Brunauer–Emmett–Teller method for specific surface area
**BJH**	Barrett–Joyner–Halenda method for pore size distribution
**XRD**	X-ray diffraction (crystallinity)
**FTIR**	Fourier-transform infrared spectroscopy (surface chemistry)
**SEM/TEM**	Scanning/transmission electron microscopy
**EDX/EDS**	Energy-dispersive X-ray spectroscopy (elemental analysis)
**ICP-MS**	Inductively coupled plasma mass spectrometry (trace purity)
**DLS**	Dynamic light scattering (particle size distribution)
**TGA**	Thermogravimetric analysis (thermal stability, organic loading)
**AI/ML**	Artificial intelligence/machine learning for synthesis optimization
**PEG**	Poly(ethylene glycol); surface grafting is commonly termed PEGylation
**SAXS**	Small-angle X-ray scattering
**XPS**	X-ray photoelectron spectroscopy
**XRF**	X-ray fluorescence spectroscopy
**ICP-OES**	Inductively coupled plasma optical emission spectrometry
**CTAB**	Cetyltrimethylammonium bromide, a cationic surfactant/template
